# Comprehensive Analysis of a Six-Gene Signature Predicting Survival and Immune Infiltration of Liposarcoma Patients and Deciphering Its Therapeutic Significance

**DOI:** 10.3390/ijms25147792

**Published:** 2024-07-16

**Authors:** Jiayang Han, Binbin Zhao, Xu Han, Tiantian Sun, Man Yue, Mengwen Hou, Jialin Wu, Mengjie Tu, Yang An

**Affiliations:** 1 Department of Biochemistry and Molecular Biology, School of Basic Medical Sciences, Henan University, Kaifeng 475004, China; 2 Henan Provincial Engineering Center for Tumor Molecular Medicine, Kaifeng Key Laboratory of Cell Signal Transduction, Henan University, Kaifeng 475004, China

**Keywords:** liposarcoma, gene signature, distant recurrence-free survival, prognostic analysis, therapeutic target

## Abstract

Background: As a common soft tissue sarcoma, liposarcoma (LPS) is a heterogeneous malignant tumor derived from adipose tissue. Due to the high risk of metastasis and recurrence, the prognosis of LPS remains unfavorable. To improve clinical treatment, a robust risk prediction model is essential to evaluate the prognosis of LPS patients. Methods: By comprehensive analysis of data derived from GEO datasets, differentially expressed genes (DEGs) were obtained. Univariate and Lasso Cox regressions were subsequently employed to reveal distant recurrence-free survival (DRFS)-associated DEGs and develop a prognostic gene signature, which was assessed by Kaplan–Meier survival and ROC curve. GSEA and immune infiltration analyses were conducted to illuminate molecular mechanisms and immune correlations of this model in LPS progression. Furthermore, a correlation analysis was involved to decipher the therapeutic significance of this model for LPS. Results: A six-gene signature was developed to predict DRFS of LPS patients and showed higher precision performance in more aggressive LPS subtypes. Then, a nomogram was further established for clinical application based on this risk model. Via GSEA, the high-risk group was significantly enriched in cell cycle-related pathways. In the LPS microenvironment, neutrophils, memory B cells and resting mast cells exhibited significant differences in cell abundance between high-risk and low-risk patients. Moreover, this model was significantly correlated with therapeutic targets. Conclusion: A prognostic six-gene signature was developed and significantly associated with cell cycle pathways and therapeutic target genes, which could provide new insights into risk assessment of LPS progression and therapeutic strategies for LPS patients to improve their prognosis.

## 1. Introduction

Liposarcoma (LPS) is a malignant mesenchymal tumor with a poor prognosis and accounts for approximately 15% of patients with sarcomas [1,2]. LPS often occurs in limbs and retroperitoneum with equal possibility, and also in the mediastinum, head and neck [3]. Based on pathological features, LPS can be classified into five main subtypes, including well-differentiated LPS (WDLPS, also known as atypical lipomatous neoplasm), dedifferentiated LPS (DDLPS), myxoid LPS (MLPS), round-cell LPS (RCLPS) and pleomorphic LPS (PLPS) [4]. Additionally, MLPS and RCLPS are normally considered as one category of LPS called myxoid/round-cell LPS (MRCLPS). WDLPS and DDLPS are more common than other subtypes [5], both of which are developed from normal adipose tissue; the former is similar to mature and variably pleomorphic adipocytes histologically, while DDLPS is mainly developed from WDLPS with additional chromosomal abnormalities and more malignant behavior, deciphering the reason why DDLPS lacks most features resembling adipose tissue [5]. Histologically, MLPS appears as non-adipocytic mesenchymal tumor cells with immature lipoblasts, RCLPS is regarded as a special variant of MLPS [5], and PLPS consists of multiple irregular cell groups and isolated cells with some features of adipocytes [6]. The prognosis of LPS varies according to its subtype classification: WDLPS or low-grade MLPS patients exhibit a higher five-year survival rate of up to 90%; conversely, that of DDLPS or PLPS could be as low as 30% [7]. Meanwhile, in MLPS, the higher the proportion of RCLPS, the worse the prognosis observed [6].

Due to its complex histopathological subtypes and high heterogeneity, the responses of LPS patients to current treatment approaches differ a lot [8]. In recent decades, LPS treatment mainly depends on surgical resection to improve the curative possibility, making adjuvant or neoadjuvant radiotherapy a supportive option [9]. Currently, chemotherapeutics, including eribulin, doxorubicin and trabectedin, have been proven to be better responses in different LPS subtypes [10,11,12,13]. However, these treatment approaches for advanced LPS patients are limited and unsatisfactory. Palliative chemotherapy, which may prolong the overall survival of patients, is usually considered as a therapy method [14]. Whereas for patients with WDLPS and DDLPS, the response to chemotherapy is not optimistic [8].

The pathogenesis of LPS has not been well-elucidated yet. So far, oncogenes, including MDM2, CDK4 and HMGA2, as well as signaling pathways, including MAPK, erbB, JAK-STAT and Wnt, may play major roles in tumorigenesis [15,16,17]. Targeted therapies, as a more specific tumor treatment against the etiology at the molecular level, have achieved relatively satisfactory curative effects in a variety of tumors. For LPS, as research on LPS-targeted therapies is ongoing, several therapeutic approaches have been demonstrated and further investigated, including co-targeting of MDM2 and CDK4/6 with siremadlin and ribociclib [18]. In view of this, it is essential to explore the underlying mechanisms of the pathogenesis of LPS and develop molecular-targeted therapeutic strategies for LPS.

During the process of disease research, it is of great significance to identify gene signatures that effectively predict the prognosis of patients, not only revealing the potential pathogenic implications of tumors and prognostic risk but also serving as potential targets for targeted therapy to resist relapse and progression of tumors at the molecular level. Therefore, this study focuses on the establishment of a prognostic risk assessment model for LPS. Here, a novel multi-gene prognostic signature of LPS was identified through machine learning, not only providing a potential method for clinical management and treatment with its robust prognostic prediction ability but also serving as a potential candidate for molecular targeted therapy of LPS. Our findings might provide foundational clinical implications for precision medicine as well as research efforts on LPS.

## 2. Results

### 2.1. Identification and Evaluation of DEGs between LPS and Normal Adipose Tissues to Elucidate the Molecular Characteristics of LPS Pathology

**Figure 1:**
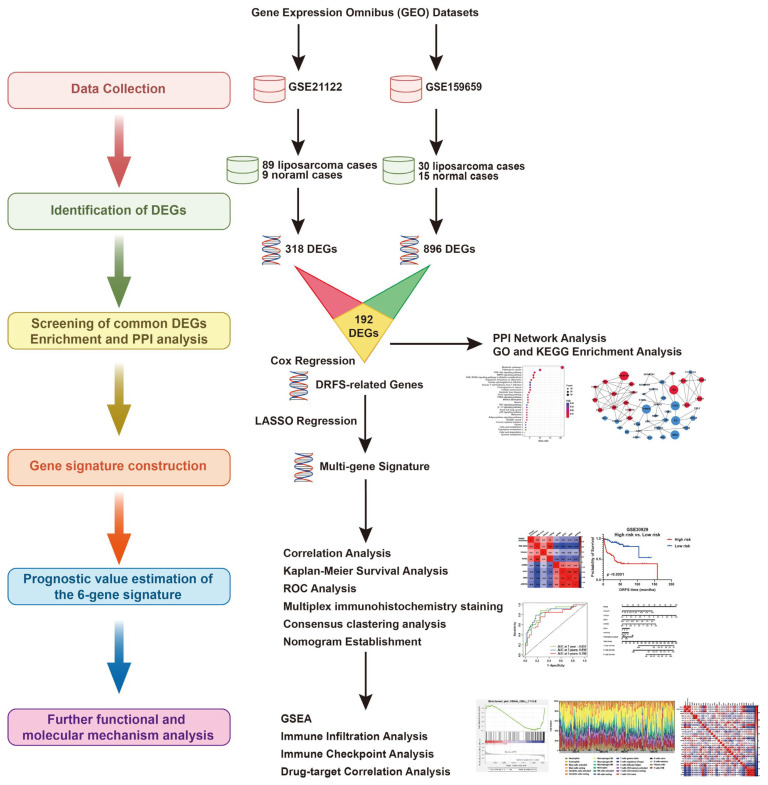
**The research flowchart of this study**.

**Figure 2:**
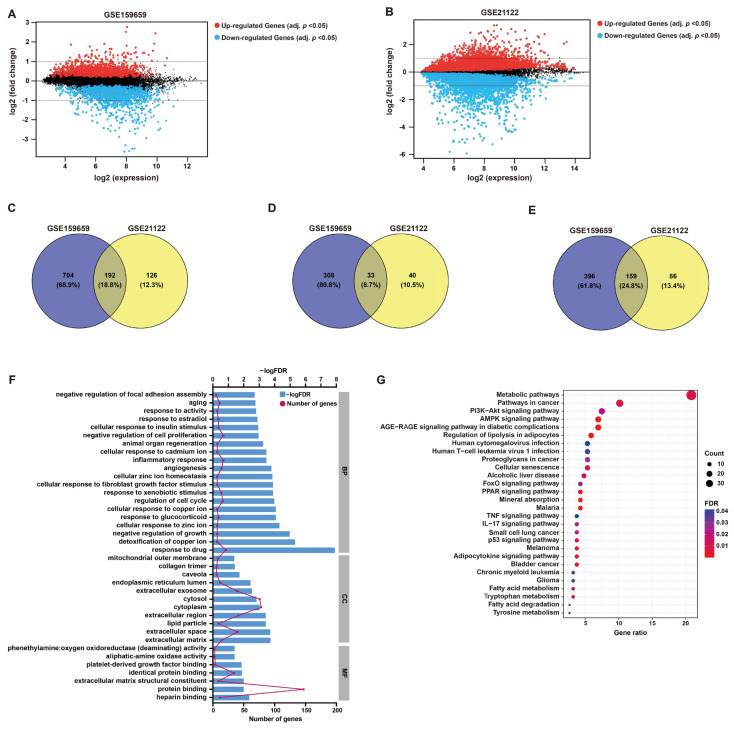
**DEG screening and enrichment analysis.** (**A**,**B**) Mean difference (MD) plots generated from GEO2R analysis show the relationship between log2 fold changes and average log2 expression values to visualize DEGs of GSE159659 and GSE21122 datasets. As shown, genes with an adjusted *p*-value cutoff of 0.05 were highlighted for their differential expression (red, up-regulated; blue, down-regulated). (**C**–**E**) Venn plots illustrate 192 overlapped DEGs (common DEGs), including 33 co-upregulated DEGs and 159 co-downregulated DEGs. (**F**) GO enrichment analysis of these common DEGs in BP, CC and MF. (**G**) KEGG pathway analysis of these common DEGs.

After the normalization of microarray data, DEGs between LPS and normal adipose tissues were identified through GEO2R analysis from two datasets (GSE21122 and GSE159659), respectively (Figure 1 and Figure 2A,B). Then, 192 common DEGs overlapped from these two datasets were selected, including 33 up-regulated and 159 down-regulated genes (Figure 1 and Figure 2C–E).

GO functional annotation and KEGG pathway enrichment were employed to investigate the potential functions of these DEGs, which may be implicated in LPS pathology. In terms of BP, the enriched functional items were associated with responses to cell growth, including “negative regulation of cell proliferation” (gene count = 17, *p* < 0.001) and “regulation of cell cycle” (gene count = 15, *p* < 0.001) (Figure 2F), which refer to the characteristics of tumor cells. Additionally, items “inflammatory response” (gene count = 17, *p* < 0.001) and “response to drug” (gene count = 21, *p* < 0.001) were also functionally enriched (Figure 2F). The functional items of MF were clustered in “protein binding” (Figure 2F). KEGG analysis showed that “metabolic pathways” (gene count = 39, *p* < 0.001), “pathways in cancer” (gene count = 19, *p* < 0.001) and “AMPK signaling” (gene count = 13, *p* < 0.001) were significantly enriched, which may contribute to the growth regulation or responses of tumor cells to drug or inflammation (Figure 2G). The detailed descriptions of GO and KEGG analyses are listed in Table S1.

Furthermore, the PPI network of DEGs was constructed using STRING and Cytoscape with a confidence level of 0.9 (Figure S1A). The top 25 candidate hub genes were presented in the network (confidence degree = 0.4) (Figure S1B). Subsequently, MCODE and ClueGO analyses were conducted to find the significant clustering modules and their potential functions (Figure S1C–E). Then the top three clustering modules were selected (Figure S2), and the network diagram of the pathways was consistent with the corresponding bar charts. Module 1 with a score of 11.492 was related to “platelet-derived growth factor binding”, “fibrillar collagen trimer”, “banded collagen fibril” and other processes (Figure S2A). Module 2, with a score of 9.308, was in connection with “response to vitamin E”, “response to hyperoxia” and other processes (Figure S2B). Module 3, with a score of 9.3, was relevant to “negative regulation of G1/S transition of mitotic cell cycle” and “negative regulation of cell cycle G1/S phase transition” (Figure S2C). More details are listed in Table S2. These findings were consistent with the results of the GO analysis of DEGs.

### 2.2. Development of a Six-Gene Prognostic Signature by Screening of DRFS-Related DEGs

**Figure 3:**
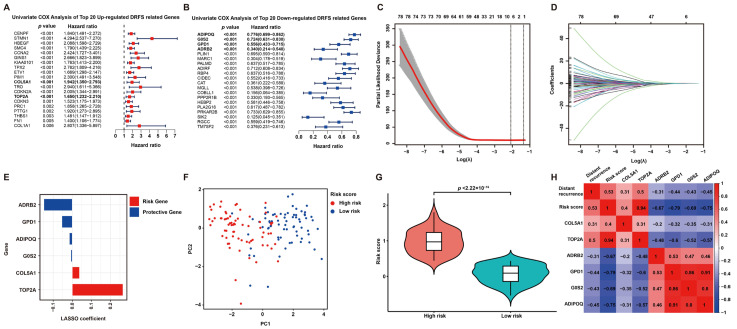
**Prognostic DEG identification and gene signature establishment.** (**A**,**B**) Significance and hazard ratio values of the top 20 up- and down-regulated DRFS-associated genes in univariate Cox analysis, respectively. Bold highlighted genes involved in the prognostic model for LPS display a significant association with DRFS. (**C**) Cross-validation for adjusting parameters for the Lasso model. Lambda.min (0.1242) and lambda.1se are shown by two black dashed lines separately, and the former indicates the six genes eventually identified. (**D**) Distribution of Lasso coefficients for DRFS-related genes obtained from univariate Cox regression. The larger the λ value, the smaller the coefficient distribution. (**E**) Lasso coefficient of the six prognostic genes derived from Lasso Cox regression. (**F**) PCA plot of patients divided into high- and low-risk groups by the median of risk scores. (**G**) Violin plot shows the significant difference in risk scores between high- and low-risk patients. (**H**) Correlation heatmap of risk score, gene expression and distant recurrence tendency contained in the six-gene signature.

To screen DRFS-related DEGs, Cox regression analysis was performed using expression profiling and survival data of 140 patients from the GSE30929 dataset. According to the univariate Cox regression analysis, 126 DEGs were found to be highly related to DRFS (Figure 3A,B). Furthermore, Lasso Cox regression was applied to construct a prognostic risk model based on these 126 DRFS-related DEGs, which consisted of six genes, including Collagen type V alpha 1 chain (COL5A1), DNA topoisomerase II alpha (TOP2A), glycerol-3-phosphate dehydrogenase 1 (GPD1), adrenoceptor beta 2 (ADRB2), G0/G1 switch 2 (G0S2) and adiponectin (ADIPOQ). The optimal value of λ and the coefficients of these six genes were illustrated, respectively (Figure 3C,D). The up-regulated genes, COL5A1 and TOP2A, with HR > 1, were regarded as LPS prognostic risk factors, while the down-regulated genes, including GPD1, ADRB2, G0S2 and ADIPOQ, with HR < 1, were considered as protective genes (Figure 3A,B,E).

According to the coefficients of Lasso Cox regression, the risk score of this signature can be calculated as follows: risk score = expression value of COL5A1*0.037947807 + expression value of TOP2A*0.265638452 + expression value of GPD1*(−0.054047643) + expression value of ADRB2*(−0.148509823) + expression value of G0S2*(−0.006938013) + expression value of ADIPOQ*(−0.014699818). Then, patients were categorized into two subgroups according to the risk score, defined as the high-risk and low-risk groups. As expected, the high-risk and low-risk patients exhibited a clear distinction, and the high-risk group held a significantly higher risk score (Figure 3F,G). Furthermore, a correlation analysis among distant recurrence probability, risk score and six signature genes was conducted to reveal a strong relevance between them (Figure 3H). In particular, the risk score or six signature genes showed a significant correlation with distant recurrence tendency; risk genes displayed a positive correlation with each other but a negative correlation with protective genes, while the results of protective genes were opposite (Figure 3H).

### 2.3. The Expression Features and Prognostic Values of the Six Signature Genes

**Figure 4:**
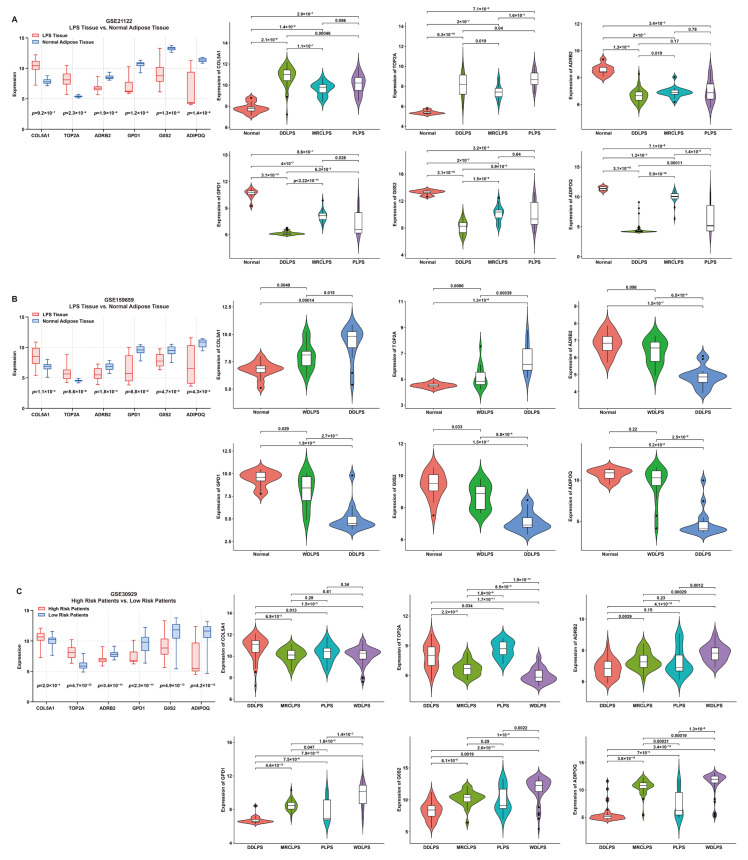
**Expression features of six signature genes involved in the risk model.** (**A**,**B**) Comparison of gene expression between LPS and normal adipose tissue (box plot) and elucidation of expression features (violin plots) of each gene from patients in GSE21122 and GSE159659 datasets, respectively. (**C**) Comparison of gene expression between low- and high-risk patients (box plot) and expression features (violin plots) of each gene from patients in the GSE30929 dataset.

The expression features of these signature genes were investigated by comparing their expression between tumor and normal tissues (Figure 4). For risk genes, compared to normal tissue, the expression of COL5A1 or TOP2A was significantly higher in LPS; on the contrary, for protective genes, the expression of ADRB2, GPD1, G0S2 or ADIPOQ was significantly higher in normal tissue than that in LPS (Figure 4A,B). Additionally, the expression of risk genes was significantly higher in high-risk LPS patients compared to the low-risk group, while the result of protective genes was the opposite (Figure 4C). Furthermore, the comparison of normal tissues with different LPS histologic subtypes revealed significant differences in expression features between risk and protective genes. As is known, DDLPS is more malignant than WDLPS. In comparison with WDLPS, patients afflicted with DDLPS exhibited significantly higher expression of risk genes but lower protective genes (Figure 4A–C), indicating that these genes might be involved in liposarcomagenesis and development.

To verify the expression features of these signature genes, multiple immunohistochemistry staining was performed. Consistent with the above results, the expression of risk genes (COL5A1 and TOP2A) in LPS tissue was significantly higher than that in normal adipose tissue, while the opposite was true for the protective genes, G0S2, GPD1, ADIPOQ and ADRB2 (Figure S3).

**Figure 5:**
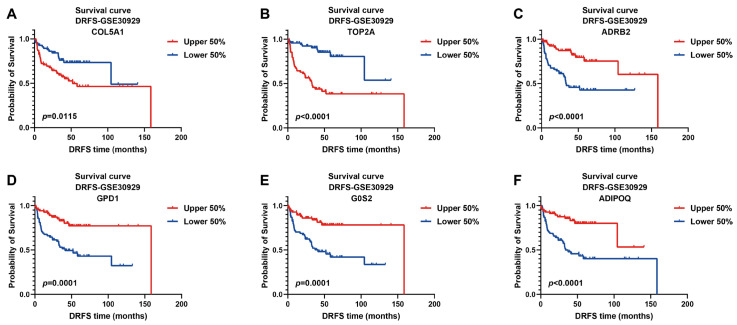
**Kaplan–Meier survival curves of DRFS for LPS patients based on the expression of six signature genes.** Two risk genes: COL5A1 and TOP2A; four protective genes: ADRB2, GPD1, G0S2 and ADIPOQ; cutoff value of 0.5; DRFS, distant recurrence-free survival.

The prognostic values of these signature genes were estimated by plotting Kaplan–Meier survival curves using DRFS data from GSE30929. As illustrated, patients with high expression of risk genes were significantly associated with worse DRFS (COL5A1, *p* = 0.0115; TOP2A, *p* < 0.0001, Figure 5A,B), indicating that they act as prognostic risk factors implicated in LPS progression. On the contrary, high expression of protective genes predicted better DRFS of patients (ADRB2, *p* < 0.0001; GPD1, *p* = 0.0001; G0S2, *p* = 0.0001; ADIPOQ, *p* < 0.0001, Figure 5C–F), implying their protective benefit to LPS prognosis.

### 2.4. Evaluation of the Prognostic Ability of Six-Gene Signature Indicates That It Is an Independent Predictor for LPS Prognosis

**Figure 6:**
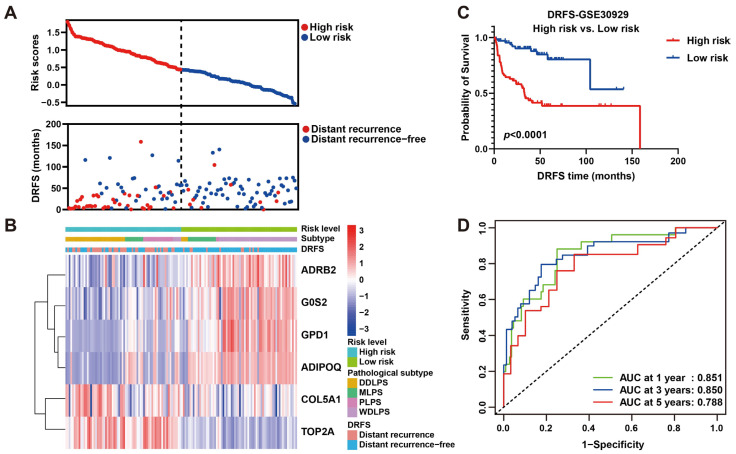
**Prognostic value estimation of the six-gene signature for LPS.** (**A**) Scatter diagrams visualize risk scores (upper) and DRFS (lower) of patients in the GSE30929 dataset. (**B**) A heat map of gene expression illustrates risk level, histological subtype LPS and DRFS status of patients ranked by risk score from the highest to the lowest. (**C**) Kaplan–Meier survival analysis of patients divided into high- and low-risk groups by the median of risk score. (**D**) ROC curves of 1-, 3- and 5-year DRFS with AUC values for predicting the efficacy of this risk model.

As shown, the number of patients who suffered from LPS recurrence in the high-risk group was more than that in the low-risk group (Figure 6A), implying this risk model may be an indicator for LPS distant recurrence. As a heat map with clinicopathological characteristics illustrated, the expression of four protective genes was higher in low-risk patients who suffered from WDLPS and lower distant recurrence, while high-risk patients who suffered from DDLPS and higher distant recurrence exhibited higher expression of two risk genes (Figure 6B), indicating a specific relation of risk score and clinicopathological characteristics. To further verify the prognostic ability of this risk model, a survival curve plotted by dividing patients into high- and low-risk groups revealed that DRFS of low-risk patients was significantly better than that of high-risk patients (*p* < 0.0001, Figure 6C), indicating the risk prediction ability of this model to distant recurrence. Subsequently, ROC curves were plotted to reveal the precision of this model in 1-, 3- and 5-year DRFS prediction for LPS (AUC values: 0.851, 0.850 and 0.788), implying the robust prognostic performance (Figure 6D). These results were validated by plotting survival and ROC curves using the validation set GSE159848, proving the strong predictive performance of this model (Figure S4). Intriguingly, our six-gene model displayed better precision in more aggressive LPS histological subtypes, including DDLPS (AUC at 1 year = 0.753, AUC at 3 years = 0.823, AUC at 5 years = 0.667), MRCLPS (AUC at 1 year = 0.945, AUC at 3 years = 0.887, AUC at 5 years = 0.920) and PLPS (AUC at 1 year = 0.702, AUC at 3 years = 0.723, AUC at 5 years = 0.724) (Figure S5). Accordingly, survival curves also revealed the significance of differences in DRFS between high- and low-risk patients in more malignant subtypes (DDLPS, MRCLPS and PLPS) (Figure S5), indicating the potential relevance of this six-gene signature to LPS progression.

**Figure 7:**
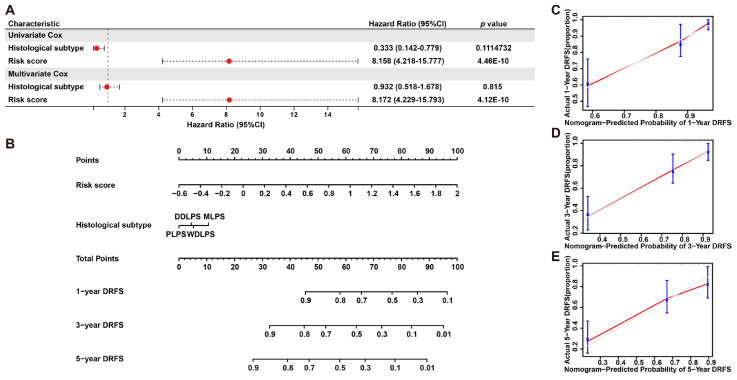
**Nomogram predicting 1-, 3- and 5-year DRFS for LPS patients based on histological subtype and risk score.** (**A**) Cox regression analysis of histological subtype and risk score in LPS. (**B**) A nomogram of 1-, 3- and 5-year estimates of the probabilities of DRFS of LPS patients. (**C**–**E**) Calibration curves of 1-, 3- and 5-year DRFS predictions.

To further validate the prognostic significance of this model, a nomogram including risk score and histological subtypes was developed based on multivariate Cox regression to evaluate the probabilities of 1-, 3- and 5-year DRFS (Figure 7A,B), and this result was confirmed by calibration plots, which mediated the comparison of actual DRFS to the nomogram-predicted DRFS (Figure 7C–E). The Harrell C-index for this nomogram was 0.783 (95% CI: 0.714–0.854), denoting the reliable performance of it. In short, these results demonstrated the robust predicting ability and potential application of this six-gene signature to clinical.

### 2.5. Patients with Different Risk Scores Exhibit Distinct Molecular Subtypes and the Enriched Gene Sets

Based on the expression pattern of the six signature genes, LPS patients with molecular heterogeneity were classified into two subgroups by consensus clustering (Figure S6A,B). The expression feature of these genes was investigated between two molecular subtypes; as a result, patients of cluster 1 presented significantly higher expression of risk genes but lower expression of protective genes compared to cluster 2 (Figure S6C). Furthermore, a survival curve demonstrated the significant prognostic differences between these two molecular subtypes, as illustrated; patients of cluster 1 underwent a poor prognosis, while cluster 2 received a beneficial prognosis (Figure S6D). Moreover, a Sankey diagram revealed the corresponding relationship of molecular subtypes to risk score or histological subtypes: cluster 1 with a worse prognosis mainly corresponded to more malignant DDLPS and PLPS and mostly represented the high-risk group, while cluster 2 with a better prognosis mainly corresponded to the low-risk group. (Figure S6E), indicating the significance of this signature to determine the molecular heterogeneity of LPS.

**Figure 8:**
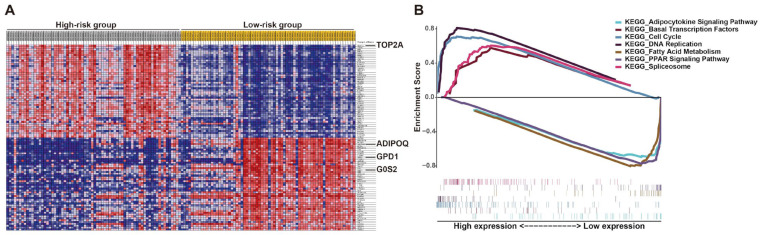
**Gene set enrichment analysis** (**GSEA**) **of LPS patients stratified by risk score.** (**A**) GSEA heat map illustrated by risk level. (**B**) Enrichment score for each pathway and corresponding gene hits.

To explore the potential molecular mechanism of the effects of this prognostic signature on LPS progression, gene set enrichment analysis (GSEA) of high- and low-risk groups was performed according to the risk score. The results revealed that the high-risk group was largely enriched in cell cycle, DNA replication, spliceosome and basal transcription factors, which were involved in cell growth (Figure 8). Fatty acid metabolism, PPAR and adipocytokine signaling were enriched in the low-risk group, which were associated with cell differentiation (Figure 8), implying cells in patients with low prognostic risk might be mostly well differentiated, consistent with the above results that the low-risk group with better prognosis mainly corresponded to WDLPS (Figure 6C and Figure S6E). These KEGG pathways enriched in the high-risk group revealed the potential molecular alterations in the malignant proliferation and aggressiveness of LPS cells in patients with high prognostic risk, indicating the involvement of this signature in liposarcomagenesis and development. The detailed outcomes of GSEA are displayed in Table S3. Additionally, the potential roles of these genes in LPS were demonstrated by single-gene GSEA (Figure S7). Interestingly, the results of single-gene GSEA analysis showed that most of them were enriched in the common pathways, including cell cycle, DNA replication and oocyte meiosis (Figure S7). This finding is consistent with previous GSEA analysis (Figure 8). More details are listed in Table S4.

### 2.6. Immune Landscape Characterization of Risk Model Reveals Significant Differences in Immune Microenvironment between High- and Low-Risk Patients

**Figure 9:**
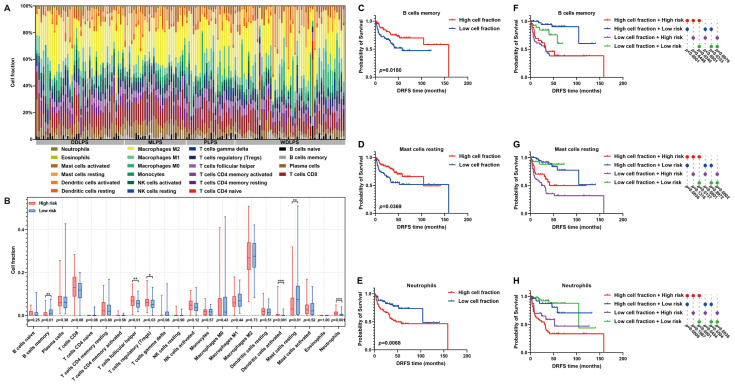
**Immune infiltration analysis of LPS TME between high- and low-risk patients.** (**A**) A histogram of immunoprofile visualizes different immune cell proportions of each patient in the GSE30929 dataset. (**B**) A box map shows cell proportion differences between high- and low-risk patients. (**C**–**E**) Kaplan–Meier survival curves illuminate the prognostic value of cell proportion of memory B cells, resting mast cells and neutrophils in LPS microenvironment. (**F**–**H**) Comprehensive Kaplan–Meier survival curves with immune cell abundance and risk score reveal the prognostic features of LPS patients. * *p* < 0.05, ** *p* < 0.01, *** *p* < 0.001.

As is known, immune cell infiltration in the tumor microenvironment (TME) is of significance to tumor progression. Thus, the immune landscape of the fraction of immune cells in each LPS sample was graphed (Figure 9A). Furthermore, the immunoprofile of high- and low-risk patients was plotted by estimating the differences in immune cell proportion between them. In detail, the proportion of memory B cells, T follicular helper cells, regulatory T cells (Tregs), activated dendritic cells, resting mast cells or neutrophils exhibited significant differences between high- and low-risk patients (Figure 9B). In particular, the infiltration of memory B cells or resting mast cells was higher in low-risk patients, while more neutrophils infiltrated in the TME of high-risk patients (Figure 9B). Furthermore, the influences of these immune characteristics on LPS prognosis were investigated. As survival curves illustrated, abundant memory B cells or resting mast cells predicted a beneficial DRFS, while abundant neutrophils denoted an adverse prognosis in LPS (Figure 9C–E). By comprehensively considering immune cell abundance and risk score, it was proved that the DRFS of low-risk patients with low proportion of memory B cells, resting mast cells or neutrophils was significantly better than that of high-risk patients with more abundant memory B cells, resting mast cells or neutrophils (*p* = 0.0340; *p* = 0.0137; *p* < 0.0001, Figure 9F–H), indicating that risk score might exert the key effect on LPS prognosis. These findings revealed the potential implications of this risk model in immune infiltration and LPS progression.

Next, the correlation of risk score with immunotherapy was estimated. In view of immune checkpoints, YTHDF1 was found to have a more significant correlation with risk scores (Figure S8A). To further explore the significance of risk score to immunotherapy, risk score was calculated in the immunotherapy datasets, and its predictive ability was assessed. As a result, high-risk patients mostly showed no response to immunotherapy (Figure S8B). In the Braun dataset, patients with higher scores exhibited a worse prognosis than those with lower scores (Figure S8C). These findings revealed the potential correlation of the risk model with the immunotherapeutic responsiveness of LPS patients.

### 2.7. Elucidation of the Therapeutic Significance of the Prognostic Signature to LPS Patients

**Figure 10:**
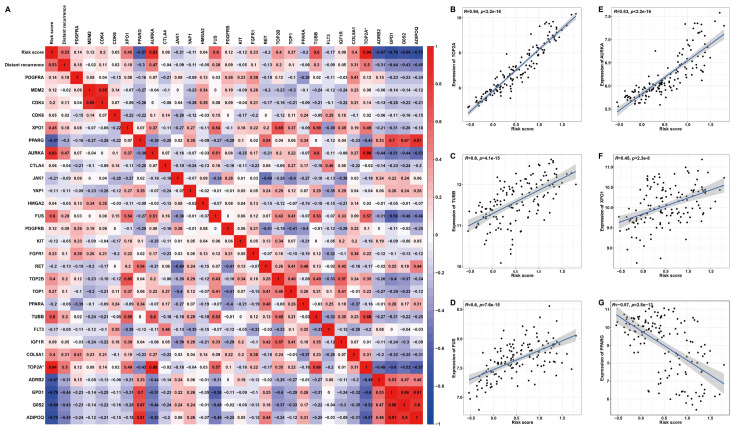
**Pearson’s correlation analysis between risk score and drug targets for LPS.** (**A**) A correlation heat map of risk score, distant recurrence, expression of target genes and six signature genes. (**B**–**G**) Scatter plots illuminate the correlation between risk score and drug target gene expression. * TOP2A is both a drug target and one of the six signature genes.

Based on previously summarized therapeutic drugs for LPS patients [19], the corresponding drug targets were employed to perform Pearson correlation analysis with the risk score (Figure 10). The heat map and scatter plots illustrated a significant positive correlation between risk score and AURKA (R = 0.83, *p* < 2.2 × 10^−16^), FUS (R = 0.6, *p* = 7.6 × 10^−15^), TOP2A (R = 0.94, *p* < 2.2 × 10^−16^), XPO1(R = 0.45, *p* = 2.3 × 10^−8^) or TUBB (R = 0.6, *p* = 4.1 × 10^−15^) (Figure 10B–F), high expression of which predicted unfavorable DRFS (Figure S9A–D). The risk score was significantly negatively correlated with PPARG (R = −0.57, *p* < 2.8 × 10^−13^) (Figure 10G), which denoted favorable DRFS (Figure S9E). Additionally, these target genes displayed distinct expression features. In detail, the expressions of TUBB, FUS, AURKA or XPO1 were significantly higher in LPS or the high-risk group compared to normal tissues or the low-risk group, while PPARG was significantly down-regulated in LPS tissues or the high-risk group (Figure S10). These results proposed a possibility that high-risk patients with up-regulated target genes, including AURKA, FUS, TOP2A, TUBB and XPO1, might be more sensitive to the corresponding chemotherapeutic drug, as the correlation of this risk model with drug targets might reveal the key role of it in enhancing the chemotherapy sensitiveness and effect. In the prospect of LPS treatment, our findings may provide a new idea for strategies of molecular targeted therapy through stratifying patients into high- and low-risk groups.

## 3. Discussion

As a common soft tissue sarcoma, despite its overall rarity, LPS has a high malignancy, leading to the poor prognosis of patients, and the incidence increases with age [20,21]. Currently, surgery is still the primary treatment option, yet the outcomes are not satisfactory due to the molecular heterogeneity, and different subtypes of LPS have distinctive gene expression patterns and clinical features, leading to the prognostic risk of different extent (high-risk patients are vulnerable to recurrence at distant sites) [1,22]. Therefore, it is of significance to investigate gene signatures for prognostic risk assessment in LPS patients and advance its application to clinical evaluation and molecular targeted therapy. Although some gene signatures for LPS have been reported, due to their limited sample size and performance to be improved, it is necessary to develop gene signatures with advanced performance using larger sample size cohorts [23]. In this study, we constructed a six-gene signature as a prognostic model, which can provide a better prognostic performance for LPS patients. In contrast to previous studies on analyzing LPS gene profiles based on microarray data, we employed the more accurate Lasso algorithm to obtain this six-gene signature, which was further investigated, evaluated and validated to make our results more reliable and prove its robustness. Compared to previous studies [23,24,25], our model displayed more robust and beneficial performance: C-index in our risk model is much higher than that in Liu’s model (0.779 vs. 0.711; 0.770 vs. 0.587); AUC value at 3-year of our risk model is much higher than that of Liu’s model (0.850 vs. 0.772) (Table 1, Figure S11).

In the construction of this model, DRFS-related DEGs were screened by univariate Cox analysis and further employed to establish a six-gene signature prognostic model through the Lasso algorithm, including four protective genes (GPD1, ADRB2, G0S2 and ADIPOQ) and two risk genes (COL5A1 and TOP2A). Collagen type V alpha 1 chain (COL5A1) is engaged in extracellular matrix collagen synthesis, and its aberrant deposition is considered as an important marker for tumor progression and metastasis [26,27]. COL5A1 was detected to be highly expressed in multiple cancers, including breast, ovarian and pancreatic cancer [28]; Topoisomerase II alpha (TOP2A) is necessary for the cell cycle and highly expressed during the mitotic process [29]. Overexpression of TOP2A occurs in a variety of cancers and participates in carcinogenesis through multiple pathways [24,30,31,32,33,34,35]. Glycerol 3-phosphate dehydrogenase 1 (GPD1) is involved in the dihydroxyacetone phosphate (DHAP) metabolic pathway and the glycerol-3-phosphate shuttle pathway [36]. GPD1 has been reported to play an anti-cancer role in a variety of cancers, including bladder, breast, lung and prostate cancers [37,38,39,40]. G0/G1 switch gene 2 (G0S2) was initially characterized according to its differential expression from the G0 to G1 phase [41]. In LPS, high dysregulation of G0S2 caused by simultaneous knockdown of adipose triglyceride lipase (ATGL) and hormone-sensitive lipase (HSL) has been proved to serve as a potential early genetic marker of this disease [42]. As a type of adipocytokine, adiponectin (encoded by ADIPOQ) can negatively regulate cancer cell growth by inducing autophagic death of cancer cells [43]. ADRB2 may participate in tumorigenesis by influencing cell proliferation, invasion and angiogenesis [44]. In short, these signature genes play different roles in the progression of cancer/tumor, advance or inhibition, corresponding to risk or protective genes. Furthermore, these DEGs were found to be implicated in cell cycle and proliferation analyzed by PPI, GO and KEGG enrichment, consistent with the GSEA results of this six-gene signature that the high-risk group was mainly enriched in cell cycle and DNA replication. For malignant tumors, it is typical that cell cycle or proliferation is accelerated by oncogene mutation, and in LPS, this is associated with the high level of amplification of the chromosome 12q1315 region, including CDK4 and MDM2 (regarded as cell cycle oncogenes) [21,45]. MDM2 facilitates tumorigenesis by inhibiting the tumor suppressor p53, and excessive activation of CDK4 leads to a cell cycle entry from the G1 to S phase and triggers unrestrained cell growth [46]. Our findings may provide an extensive understanding of the mechanisms of oncogenesis and progression of LPS.

As is known, TME has impacts on anti-tumor immunity; thus, risk score-related immune characteristics were estimated to reveal the potential immunological value of this six-gene signature. Through comparing differences in immune cell infiltration between high- and low-risk patients, memory B cells, resting mast cells and neutrophils have been found to influence DRFS of LPS patients. Previous studies confirmed that antibodies against tumor cells played an important role in anti-tumor immunity [47], and memory B cells were considered as the storage of plasma cells, which may contribute to the antibody production in secondary response [48]. Thus, memory B cells might be a protective factor in LPS progression by regulating antibody secretion by plasma cells. It has been proved that resting mast cells are capable of increasing the proliferation of B cells and their differentiation into cells, producing antibodies in tumor tissues [49], which might be regarded as a protective factor in LPS patients. However, neutrophils exhibited complicated functions in anti-tumor immunity and may suppress anti-tumor immunity in gastric cancer [50] by restraining the production of T cells and the secretion of IFN-γ, which could be the reason for its correlation with poorer DRFS in LPS patients. Significantly, in the low-risk group, patients with higher memory B cells tend to have the best DRFS, while high-risk patients with higher neutrophils are likely to have the worst DRFS, indicating these immune characteristics might help divide patients into more specific risk groups and improve their prognosis through the combined application of immunotherapy and targeted chemotherapy.

Recently, targeted therapy for anti-cancer/tumor is becoming more and more concerning. Here, we found that the risk score had a significant correlation with chemotherapy drug-targeted genes, including AURKA, FUS, TOP2A, TUBB and PPARG. It has been reported that PPARG holds a potential action in dedifferentiated LPS and acts as a tumor suppressor in many tumors [51]. Expression profiling of PPARG analyzed in three datasets showed lower PPARG in DDLPS than in normal tissues and WDLPS (Figure S10). These results are consistent with those of Wu et al., who reported that the reduction of PPARG may cause the initiation of cell proliferation and stagnation of differentiation, leading to the development of WDLPS into DDLPS [52]. As for other genes, it has been reported that FUS-DDIT3 fusion mediates IGF-IR/PI3K/AKT signaling to activate YAP1, which plays a fundamental role in cell proliferation and survival, cooperatively regulating FUS-DDIT3 targets and aiding in FUS-DDIT3-mediated block of adipocyte differentiation, ultimately leading to the development of myxoid liposarcoma [53]. Tubulin is regarded as one of the main anti-cancer targets, playing an important role in cell division and intracellular transport [54]. AURKA has been observed to be overexpressed in multiple cancers and is involved in centrosome segregation, mitotic time conditions and spindle assembly [55]. Each of these genes is associated with the development and pathogenesis of LPS. Thus, it is supposed that patients with high risk scores also have the elevated expression of AURKA, FUS, TOP2A, TUBB and XPO1 but reduced PPARG and are more sensitive to chemotherapeutic drugs, implying the key predictive role of this risk model in clinical therapy. TOP2A is both a gene in this six-gene signature risk model and a target for anti-cancer drugs.

Tumor molecular heterogeneity has been the topic of LPS research. Based on the gene expression profiles of LPS and these six gene markers, we found that the expression profiles in specific histological subtypes of LPS showed significant differences, for which the most suitable therapeutic drugs and strategies can be selected or developed to improve the therapeutic efficacy and overcome the drug resistance. The understanding of heterogeneity is also manifested in the biological manifestations, the development mechanisms and the immune microenvironment of tumors, for which more in-depth studies are needed subsequently. Our findings are concerned with the prognostic markers of LPS, which may help researchers further evaluate the risk characteristics and therapeutic responses under different tumor cell populations.

In summary, the prognostic markers we constructed may not only enrich the understanding of tumor heterogeneity but also exert effects on the diagnosis, treatment and prognostic management of LPS in clinical practice in the hope of improving the quality of patient survival and treatment success.

## 4. Materials and Methods

The flowchart of the establishment of the six-gene signature and its prognostic ability estimation is illustrated in Figure 1.

### 4.1. Acquisition of Expression Profiling Data and Clinical Information

For LPS, clinical and expression profiling data were obtained from the Gene Expression Omnibus (GEO, https://www.ncbi.nlm.nih.gov/geo/, accessed on 2 March 2022). The gene expression profiles as microarray data were downloaded from the GEO database: GSE21122, GSE159659, GSE30929 and GSE159848 (Table 2). The dataset GSE21122 [56] contains a series of 98 samples, including 89 LPS and 9 normal adipose tissue cases; the dataset GSE159659 [57] includes 30 LPS and 15 normal adipose tissue samples; the dataset GSE30929 [24] is composed of 140 LPS patients with survival data (distant recurrence-free survival, DRFS); the dataset GSE159848 [58] contains 50 LPS patients with survival data (overall survival, OS).

### 4.2. Identification of Differentially Expressed Genes by GEO2R

As an online analysis tool, GEO2R (http://www.ncbi.nlm.nih.gov/geo/geo2r/, accessed on 5 June 2022) was taken to screen differentially expressed genes (DEGs) between LPS and normal adipose tissue samples from GSE21122 or GSE159659. A DEG was defined when it matched the standards of false discovery rate (FDR) < 0.05 and |log2 fold change (FC)| ≥ 1. To further screen DEGs, the Venn maps were drawn to obtain the overlapped DEGs (common DEGs) by Venny 2.1.0 (https://bioinfogp.cnb.csic.es/tools/venny, accessed on 5 June 2022).

### 4.3. Functional Annotation and Pathway Enrichment Analysis

To further discover the underlying biological function of these DEGs, the DAVID online tool (https://david.ncifcrf.gov/) was used to carry out the enrichment analysis of Kyoto Encyclopedia of Genes and Genomes (KEGG) [59] and Gene Ontology (GO), including biological process (BP), molecular function (MF) and cellular component (CC) [60]. The *p*-values were adjusted via the FDR methodology, and a pathway was deemed to be significantly enriched when the FDR < 0.05.

### 4.4. Protein–Protein Interaction Network and Module Analysis

The protein–protein interaction (PPI) network is commonly employed to recognize the hub genes involved in the development of disease. The common DEGs were imported into the STRING database (http://string-db.org/), which is an online tool designed to analyze the PPI of genes with a confidence score ≥0.4 [61]. Then, the PPI network was constructed and visualized with Cytoscape software (version 3.8.0) [62]. Then, the maximal clique centrality (MCC) algorithm in Cytohubba was applied to calculate the hub nodes. Module analysis and GO analysis were subsequently implemented to elucidate the significantly enriched biological function of gene modules through Cytoscape plugins, MCODE and ClueGO.

### 4.5. Univariate, Multivariate and Lasso Cox Analyses

The common DEGs were selected from GSE21122 and GSE159659 to establish a model predicting distant recurrence-free survival (DRFS) of LPS patients. Univariate Cox regression analysis was applied using the “survival” package of R to ascertain the DRFS-related genes. Genes with *p* < 0.05 were defined and selected for Lasso Cox regression analysis to produce a survival-based multi-gene prognostic risk assessment model through the “glmnet” package of R. The risk level of individual patients was defined by the risk score based on the expression of the signature genes. For each patient, the risk score was the sum of the products of the corresponding gene expression and the regression coefficient. The equation is presented below:

$$Risk\;score=\sum_{i=1}^{n}{Lasso\;coefficient}_{i}\times Gene\;{expression}_{i}$$

The median risk score was applied to categorize each patient into high-risk or low-risk groups. To investigate the precision of the model, principal component analysis (PCA) and Wilcoxon rank-sum test were carried out to determine whether the performance of the genes engaged in the model could discriminate patients into the high- and low-risk groups. Subsequently, to further estimate the prognostic model and assess its prognostic significance and capacity, Kaplan–Meier (KM) survival analysis was performed, and the area under the curve (AUC) of the receiver operating characteristic (ROC) curve was calculated. The 50 samples in the GSE159848 dataset with overall survival (OS) were used for external validation.

### 4.6. Consensus Clustering Analysis for LPS

Based on prognostic genes involved in the risk model, samples from GSE30929 were clustered using the ConsensusClusterPlus package [63]. The selection criteria of clustering were that the cumulative distribution function (CDF) curve rises rapidly; the sample size of each group should not be too small after categorization; the intra-group correlation is large while the inter-group correlation is low.

### 4.7. Construction and Validation of Predictive Nomogram

In order to evaluate the probability of prognostic gene signature in LPS, all independent characteristics of patients, including the histological subtypes and genes in the risk model, were combined to construct the nomogram through a stepwise Cox regression for the purpose of predicting 1-, 3- and 5-year DRFS of LPS patients in the GSE30929 dataset. Meanwhile, Harrell’s concordance index of actual and predicted survival was calculated to evaluate the survival prediction capability and discrimination of the nomogram. The nomogram and its calibration curves were plotted by the “rms” package of R.

### 4.8. Gene Set Enrichment Analysis of Prognostic Genes

Gene set enrichment analysis (GSEA) was conducted to investigate the molecular mechanisms of LPS progression affected by the prognostic gene signature [64]. The patients from the GSE30929 dataset were classified into high- and low-risk groups by the median risk score. GSEA was performed in javaGSEA (version 4.2.3) according to the “Molecular Signature Database v. 2022.1. C2 (curated gene sets)”, which was selected to screen the enriched KEGG pathways related to survival outcome of the high- and low-risk groups. It is considered as statistically significant if |NES| > 1 and FDR q-val < 0.25.

### 4.9. Association of Risk Scores with Immune Characteristics

To assess the abundance of immune cells, the CIBERSORTx portal website (https://CIBERSORT.stanford.edu/) was applied to provide an online calculative tool with a computational method [65]. It supplies a signature file called “LM22”, a validated leukocyte gene signature matrix, which consists of 547 genes to distinguish 22 human hematopoietic cell phenotypes, including seven T cell types, B cells, plasma cells, natural killer (NK) cells and myeloid subsets. Here, the gene expression matrix data were uploaded to CIBERSORTx and ran with the default feature matrix and parameters. Subsequently, bar charts and comprehensive survival analysis with cell fractions and risk levels were plotted to visualize the final output of CIBERSORTx and examine differences between high- and low-risk groups based on the median of cell fraction or risk score by using GraphPad Prism 9.

Furthermore, the correlation of risk scores with immune checkpoints, inhibitors and stimulators in LPS patients was analyzed. GSE35640 (melanoma) [66] and Braun (renal cell carcinoma) [67] datasets were used to predict the immunotherapy response related to the risk score.

### 4.10. Exploration of the Correlation between Prognostic Risk Scores and Targets of Drug Therapy

Targeted therapies are highly significant and effective approaches for the treatment of LPS. A number of targeted agents are being developed or have been clinically applied in the last decades [19]. To predict the treatment effect using the risk score, Pearson’s correlation analysis was used to observe the relevance of the prognostic risk score to the drug targets as shown below: Platelet Derived Growth Factor Receptor Alpha (PDGFRA), MDM2 Proto-Oncogene (MDM2), Cyclin Dependent Kinase 4 (CDK4), Cyclin Dependent Kinase 6 (CDK6), Exportin 1 (XPO1), Peroxisome Proliferator Activated Receptor Gamma (PPARG), Aurora Kinase A (AURKA), Cytotoxic T-Lymphocyte Associated Protein 4 (CTLA4), Janus Kinase 1 (JAK1), Yes1 Associated Transcriptional Regulator (YAP1), High Mobility Group AT-Hook 2 (HMGA2), FUS RNA Binding Protein (FUS), Platelet Derived Growth Factor Receptor Beta (PDGFRB), KIT Proto-Oncogene, Receptor Tyrosine Kinase (KIT), Fibroblast Growth Factor Receptor 1 (FGFR1), Ret Proto-Oncogene (RET), DNA Topoisomerase II Alpha (TOP2A), DNA Topoisomerase II Beta (TOP2B), DNA Topoisomerase I (TOP1), Peroxisome Proliferator Activated Receptor Alpha (PPARA), Tubulin Beta Class I (TUBB), Fms Related Receptor Tyrosine Kinase 3 (FLT3) and Insulin Like Growth Factor 1 Receptor (IGF1R).

### 4.11. Multiple Immunohistochemistry Staining in Normal and LPS Tissues

To validate the expression differences of the model genes, multiplex immunohistochemistry was used to detect gene expression in normal and LPS tissue slices based on the fluorescence intensity. According to the manufacturer’s instructions, the slices were microwaved, and then the diluted primary antibody solution was successively added and incubated with them. The specific fluorescent dyes and conjugated secondary antibodies were subsequently added for incubation. Results were observed and photographed with a fluorescence microscope. Three images from each slide were selected randomly and analyzed using Image J 1.53q software to obtain the fluorescence intensity. Then, the differences in the fluorescence intensity of each protein between LPS and normal adipose tissue were compared by GraphPad Prism 9 software.

### 4.12. Statistical Analysis

All R program packages for statistical analysis were carried out using R software (version 4.0.4). Several R packages were utilized in our study, including “survival”, “ggplot2”, “glmnet”, “timeROC”, “rms” and their auxiliary packages. KM curve analysis and the plotting of the heatmap were performed by GraphPad Prism 9. Additionally, the networks and GSEA analysis were performed by Cytoscape version 3.8.0 and javaGSEA version 4.2.3. *p* < 0.05 was considered to be statistically significant.

## 5. Conclusions

A prognostic six-gene signature, composed of COL5A1, TOP2A, GPD1, ADRB2, G0S2 and ADIPOQ, was developed and investigated through a machine learning algorithm, which was significantly associated with cell cycle-related pathways, immune cell infiltration and therapeutic target genes, including AURKA, FUS, TOP2A, TUBB and PPARG. Our findings, which illustrated more evidence of tissue-specific tumorigenesis by establishing a novel six-gene signature based on DEGs, provide new insights into the risk assessment of LPS progression and molecularly targeted therapeutic strategies for LPS patients to improve their prognosis.

## Figures and Tables

**Table 1:** Comparison of our results with previous studies.

	Iura et al. [25]	Gobble et al. [24]	Liu et al. [23]	Our Study
DEG identification	Yes	Yes	Yes	Yes
GO analysis	No	No	Yes	Yes
KEGG analysis	No	Yes	Yes	Yes
Univariate Cox regression analysis	No	No	Yes	Yes
Multivariate Cox regression analysis	No	No	No	Yes
Lasso Cox regression analysis	No	No	No	Yes
Survival analysis	Yes	Yes	Yes	Yes
GSEA analysis	No	No	No	Yes
Immune landscape analysis	No	No	No	Yes
Cell experiment validation	Yes	Yes	No	No
Tissue experiment validation	Yes	No	No	Yes

**Table 2:** The datasets included in this study.

Dataset	Platform	Number of Samples (Tumor/Normal)	Reference
GSE21122	GPL96	98 (89/9)	Barretina J et al., 2010 [56]
GSE159659	GPL23159	45 (30/15)	Zuco V et al., 2021 [57]
GSE30929	GPL96	140	Gobble RM et al., 2011 [24]
GSE159848	GPL6480	50	Darbo E et al., 2023 [58]

## Data Availability

Publicly available datasets were analyzed in this study. These data can be found here: GEO database (https://www.ncbi.nlm.nih.gov/gds/?term=).

## Supplementary Materials

The following supporting information can be downloaded at: https://www.mdpi.com/article/10.3390/ijms25147792/s1.
