# Supplementary material for: Comprehensive Analysis of a Six-Gene Signature Predicting Survival and Immune Infiltration of Liposarcoma Patients and Deciphering Its Therapeutic Significance
Source: Int J Mol Sci. 2024 Jul 16;25(14):7792. doi: 10.3390/ijms25147792 (PMC11277418; doi:10.3390/ijms25147792)
Supplement: Supplementary file 1 [file ijms-25-07792-s001.zip › ijms-3051575-supplementary.pdf]

Supplementary Figure Legends

Figure S1. PPI network analysis of DEGs. (A) A PPI network graphed by Cytoscape software through processing the DEGs filtered by STRING with the highest degree of confidence = 0.9. Red bubbles refered to the up-regulated DEGs while the blue ones indicated the down-regulated DEGs. The size of the dots reflected the level of gene expression. (B) A network diagram of 25 genes that may play a central role obtained by MCC algorithm from Cytoscape. (C-E) The top 3 modules of all DEGs identified by MCODE plugin.

Figure S2. GO enrichment analysis of the top 3 MCODE modules. (A-C) Network diagrams of GO enrichment results of the 3 sub-networks given by MCODE via ClueGO. Each node is a representative enrichment pathway, the line of the node indicated the number of genes shared by pathways, and the color denoted the enrichment classification of the node. Details were listed in Table S2. Bubble charts for GO enrichment analysis via DAVID online tool corresponded with the network diagrams.

Figure S3. Multiple immunohistochemistry staining analysis. (A) Photographs of immunohistochemistry staining of 6 signature genes using antibodies labeled with different fluorescein. Green represented COL5A1 or ADRB2; Red refered to TOP2A or ADIPOQ; Yellow denoted G0S2 or GPD1. (B) Comparison of fluorescence intensity between LPS and normal adipose tissue. $^{*}p < 0.05$, $^{**}p < 0.01$, $^{***}p < 0.001$.

Figure S4. Validation of the prognostic 6-gene signature based on GSE159848. (A) Kaplan-Meier survival analysis of GSE159848 patients stratified into high- and low-risk group by X-tile software (upper 46% vs. lower 54%). (B) ROC curves for predicting efficiency of 3-, 4- and 5-year OS with AUC values.

Figure S5. Prognostic values of the 6-gene signature in 4 LPS histological subtypes. (A-D) Kaplan-Meier survival analysis for patients in GSE30929 dataset with different LPS histological subtypes, and patients were divided into high- and low-risk group by X-tile software (upper 47% vs. lower 53%). (E-H) ROC curves with AUC for DRFS prediction. DDLPS, dedifferentiated LPS; WDLPS, well differentiated LPS; MRCLPS, myxoid/round-cell LPS; PLPS, pleomorphic LPS.

Figure S6. Consensus clustering of LPS patients via risk score. (A) Consensus clustering result of GSE30929 samples revealed that patients were categorized into 2 molecular subtypes. (B) Consensus CDF chart revealed that k = 2 was the optimal classification of LPS patients. The increased area under the CDF curve corresponded to the enhanced number of subtypes. (C) Box plots illustrated the differences in expression of the 6 signature genes between these two clusters. (D) Kaplan-Meier survival curve of these 2 clusters. (E) Sankey diagram showed the relationship of molecular subtypes, prognostic risk level and histological subtype. ***$p < 0.001$.

Figure S7. GSEA analysis of 6 signature genes for LPS. (A-F) The enrichment pathways of ADIPOQ, ADRB2, COL5A1, G0S2, GPD1 and TOP2A were shown based on their expression, respectively. The samples were divided into high- and low-expression group according to X-tile and the heat map highlighted genes associated with enriched pathways.

Figure S8. Analysis of immune checkpoints, immunotherapy cohort and risk model. (A) Lollipop chart of immune checkpoints with risk scores. (B) Bar diagram revealed the differences in risk score between different immunotherapy response groups in GSE35640 cohort. R, responsive to immunotherapy; NR, no immunotherapy response (C, D) Survival curve differences between high- and low-risk group in the Braun cohort.

Figure S9. Survival curves of drug targeted genes for LPS in GSE30929.

Figure S10. Expression features of 5 drug targeted genes for LPS. (A, B) Comparison of gene expression between LPS and normal adipose tissue (box plot) and elucidation of expression features (violin plots) of each gene for patients in GSE21122 and GSE159659 dataset, respectively. (C) Comparison of gene expression between low- and high-risk patients (box plot) and elucidation of expression featureS (violin plots) of each gene for patients in GSE30929 dataset.

Figure S11. The comparison of risk model between our study and Liu's study. (A, E) Comparison of C-index of risk models in GSE30929 and GSE159848 dataset, respectively. (B-D, F-H) Comparison of ROC curves of risk models at 3-, 4- and 5-year in GSE30929 and GSE159848 dataset, respectively.
