# Supplementary figures and images for: Comprehensive Analysis of a Six-Gene Signature Predicting Survival and Immune Infiltration of Liposarcoma Patients and Deciphering Its Therapeutic Significance
Source: Int J Mol Sci. 2024 Jul 16;25(14):7792. doi: 10.3390/ijms25147792 (PMC11277418; doi:10.3390/ijms25147792)

A

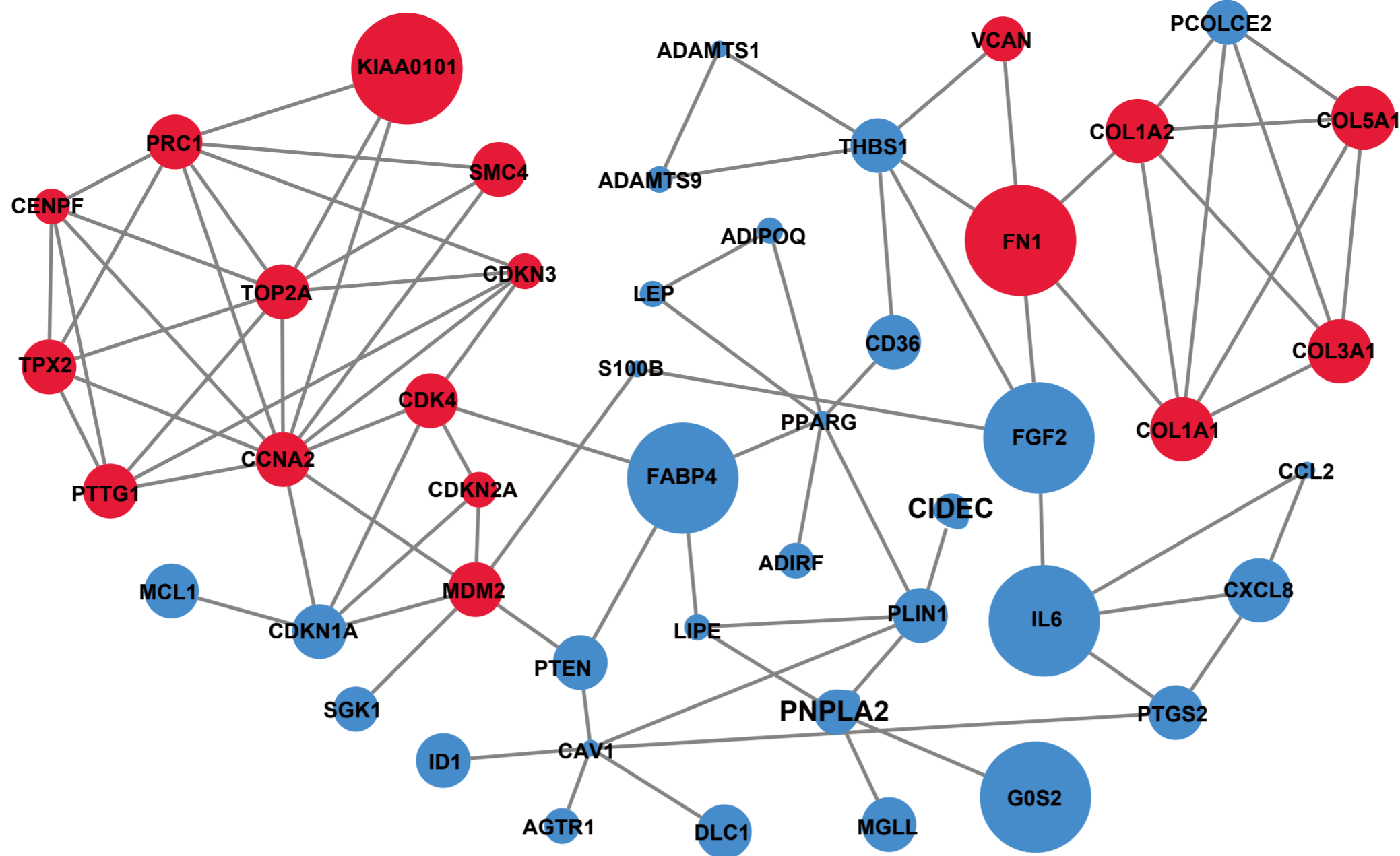

B

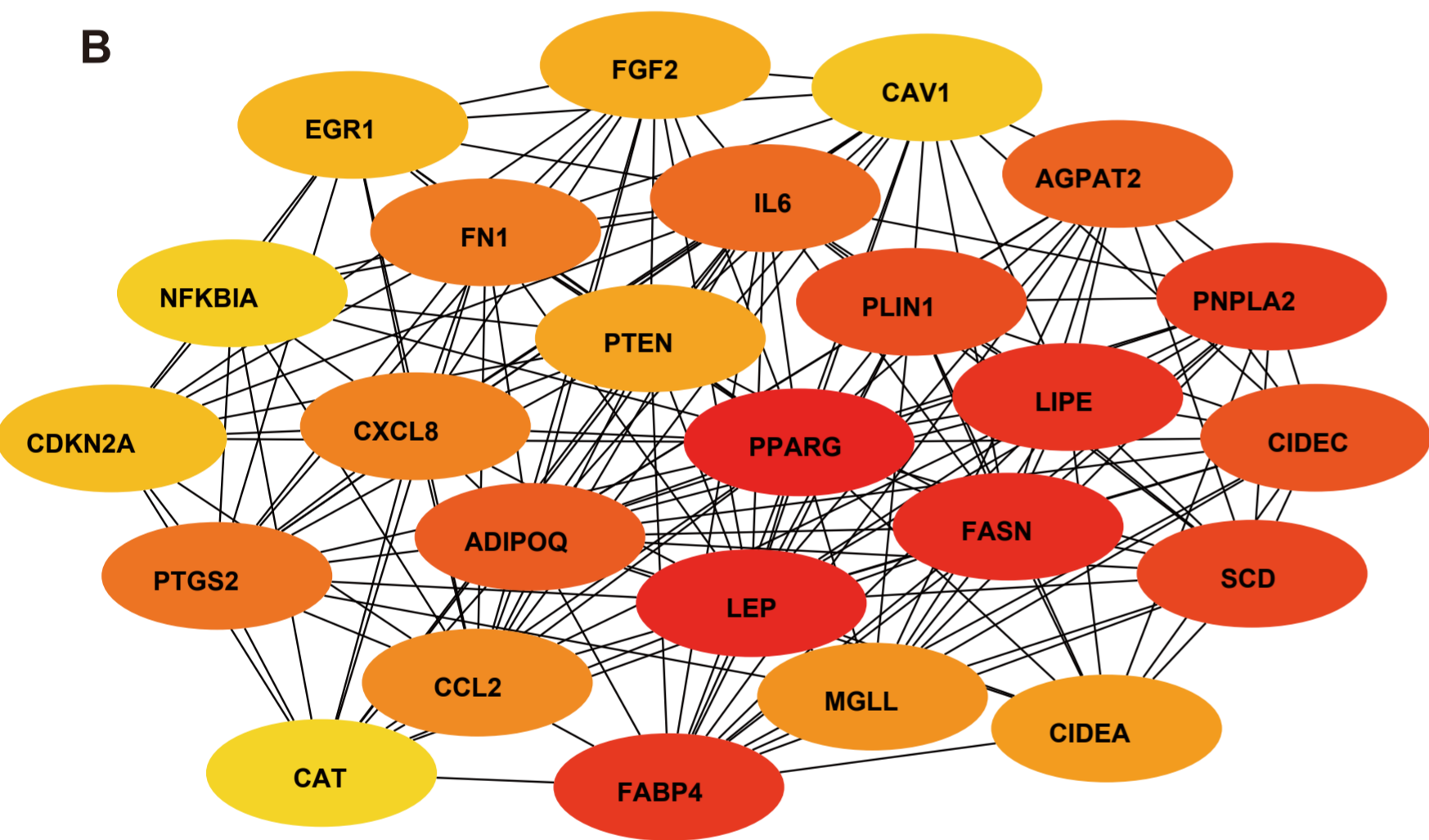

C

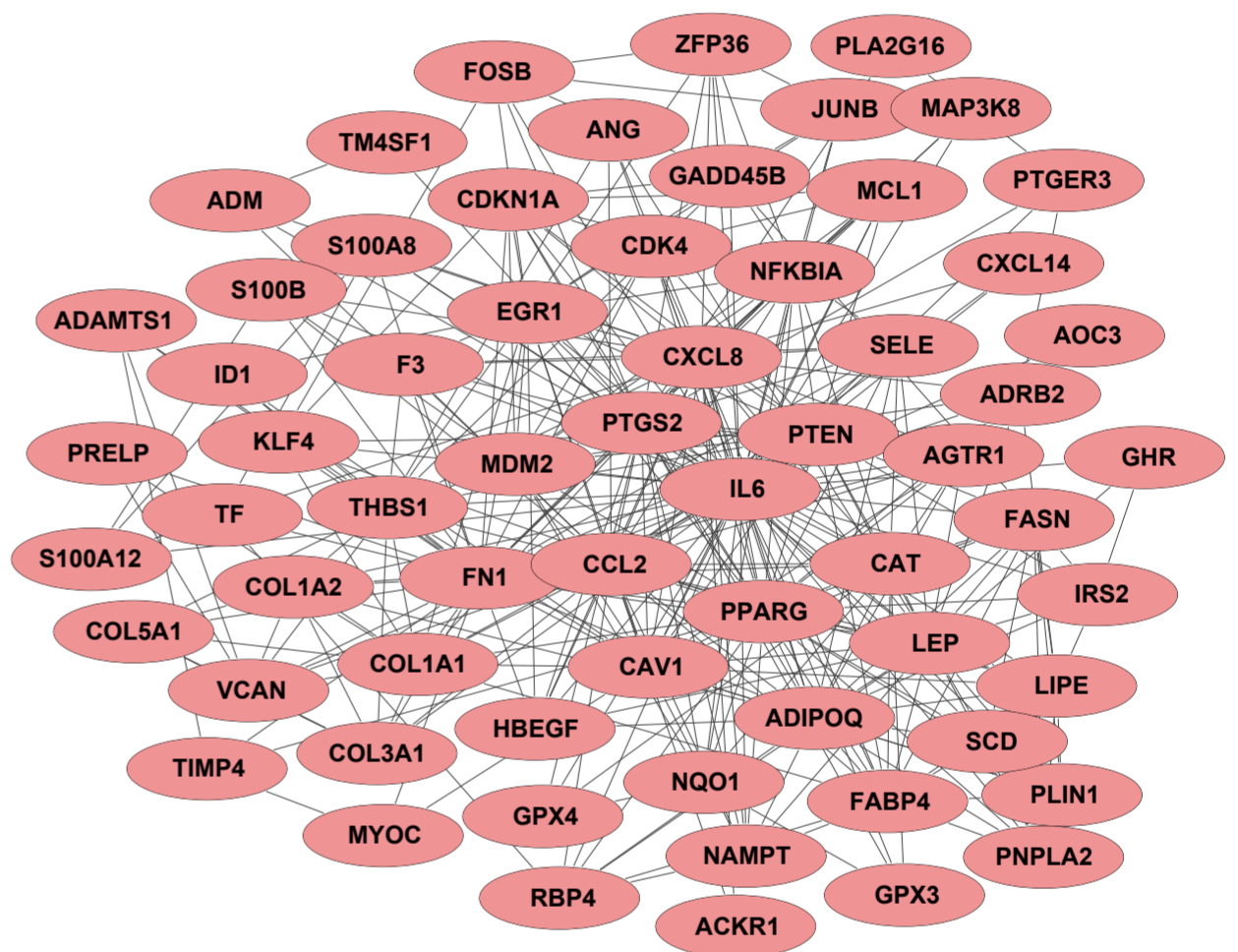

D

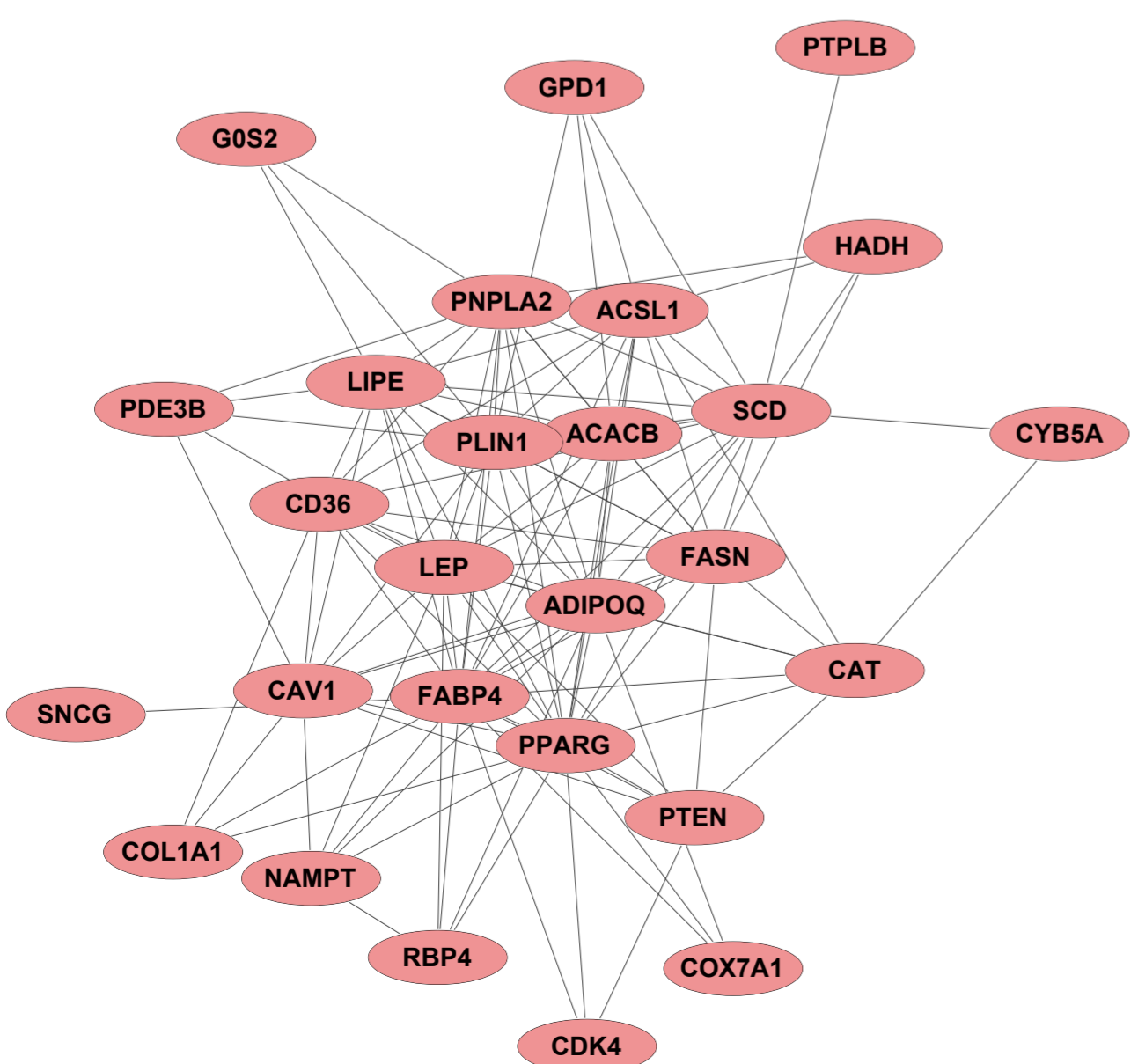

E

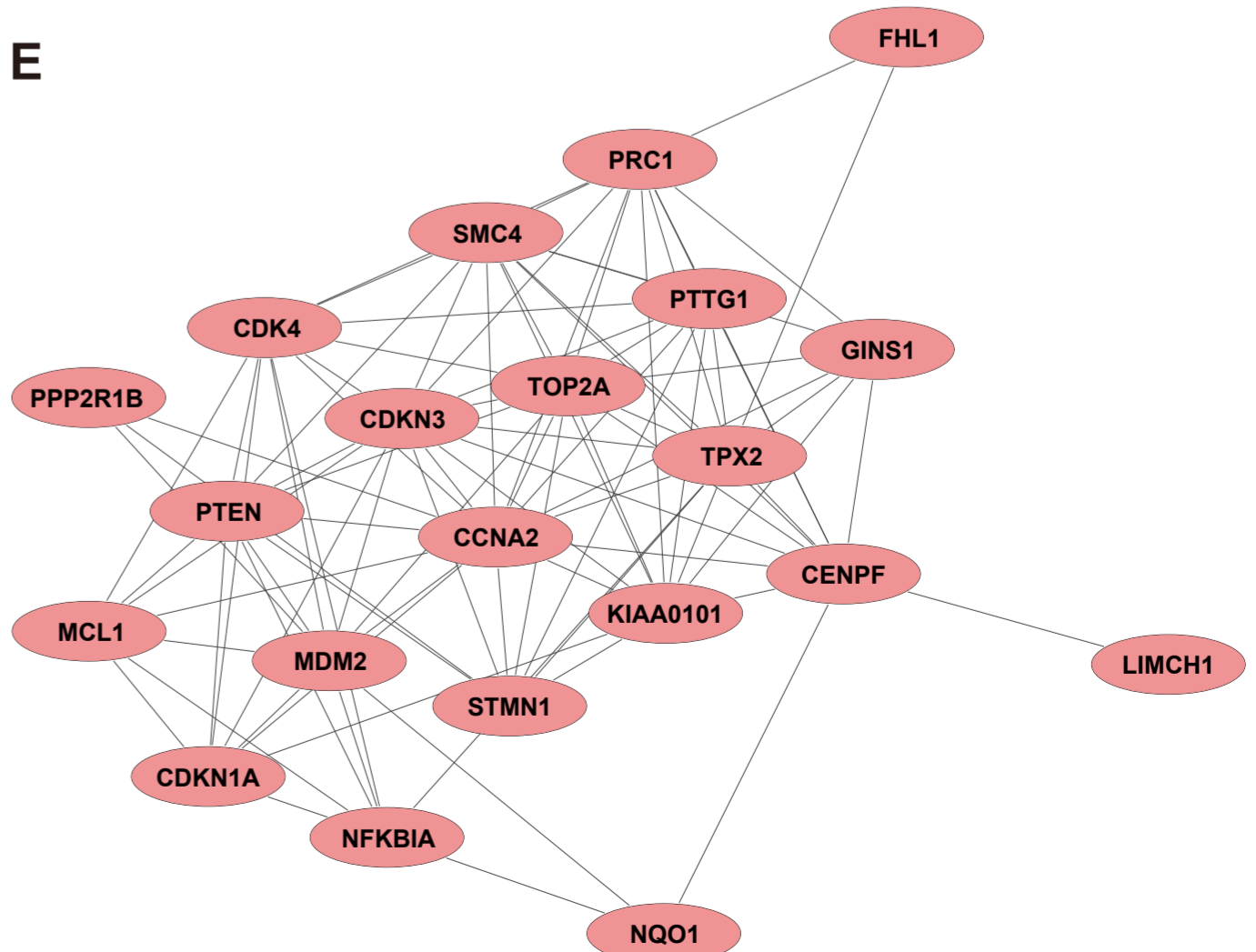

Supplement: Supplementary file 1 [file ijms-25-07792-s001.zip › Supplementary Figures/Figure S1.pdf]

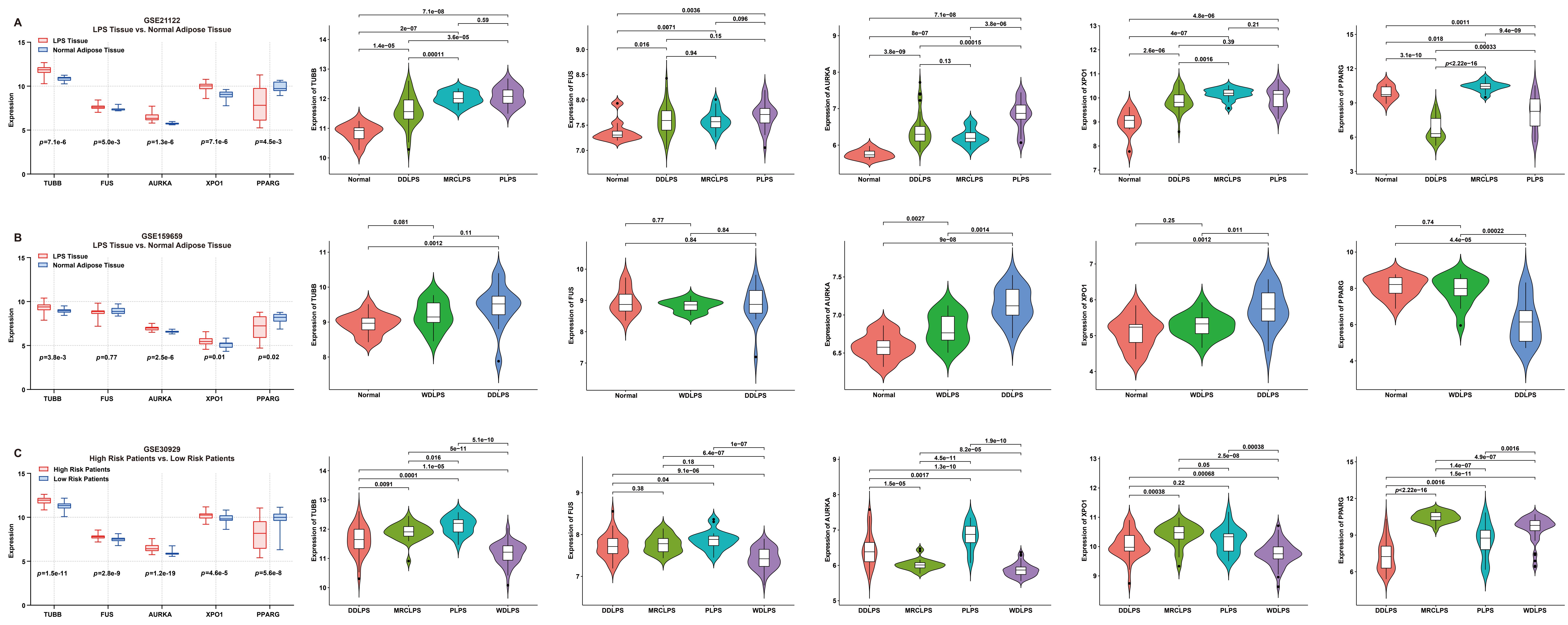

Supplement: Supplementary file 1 [file ijms-25-07792-s001.zip › Supplementary Figures/Figure S10.pdf]

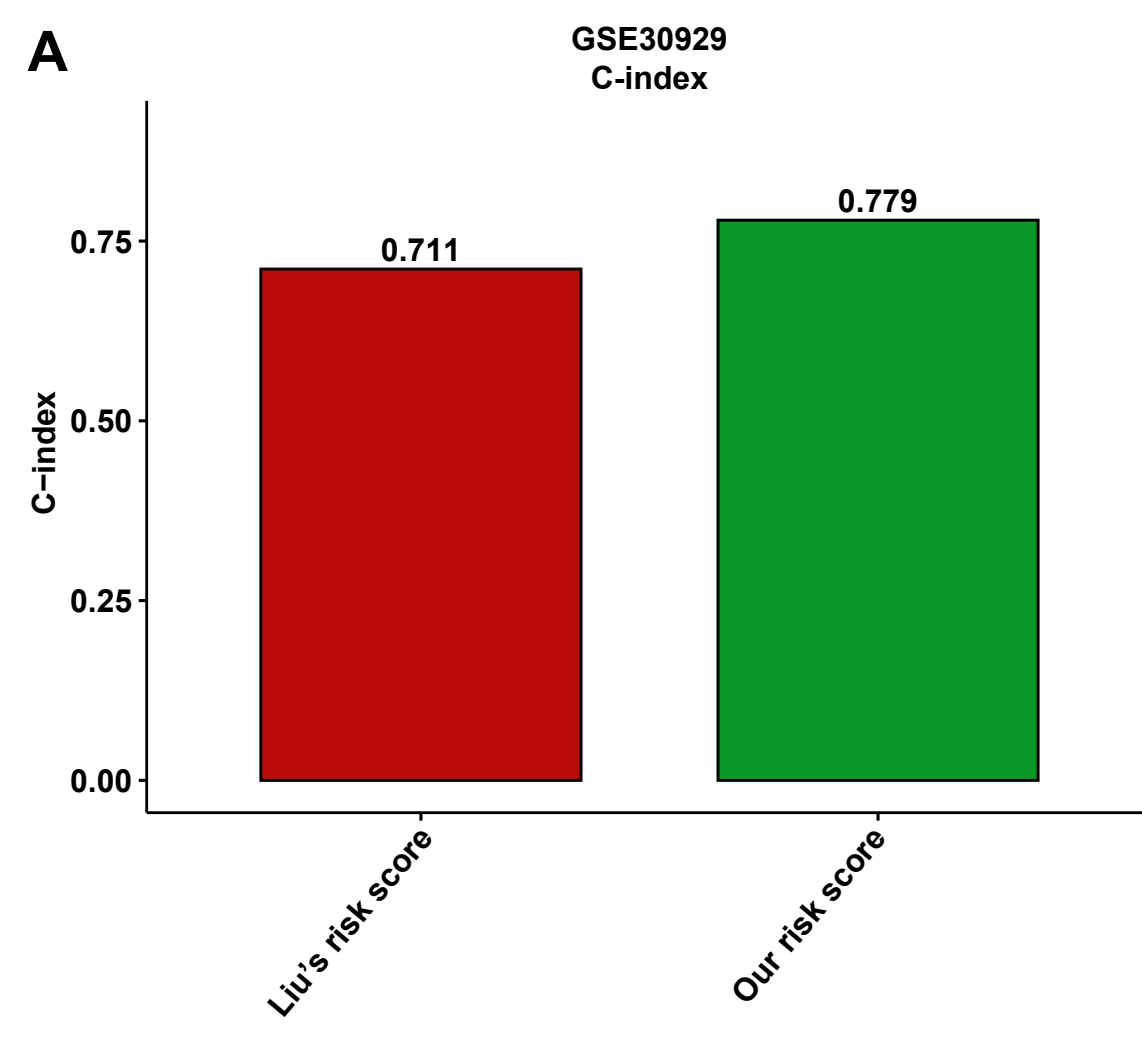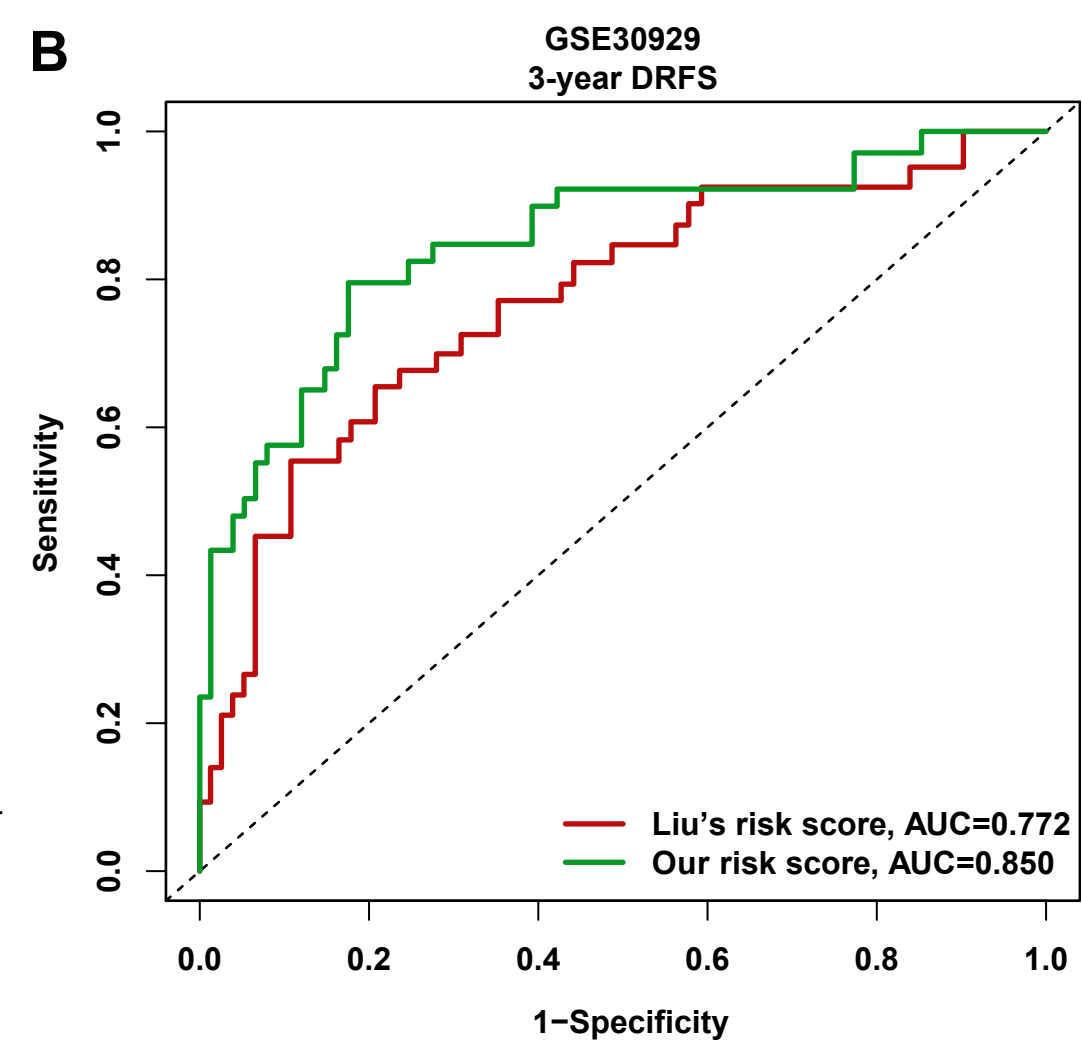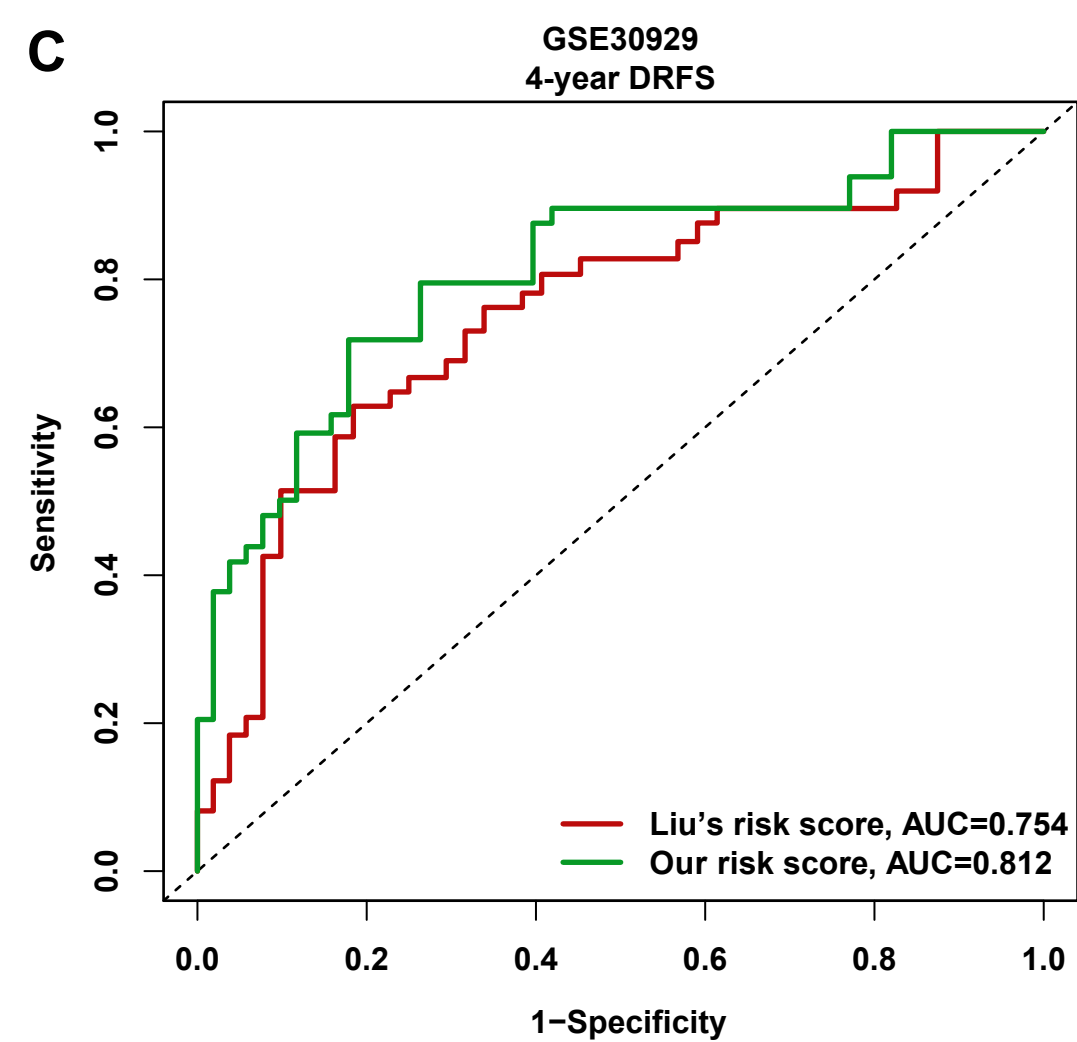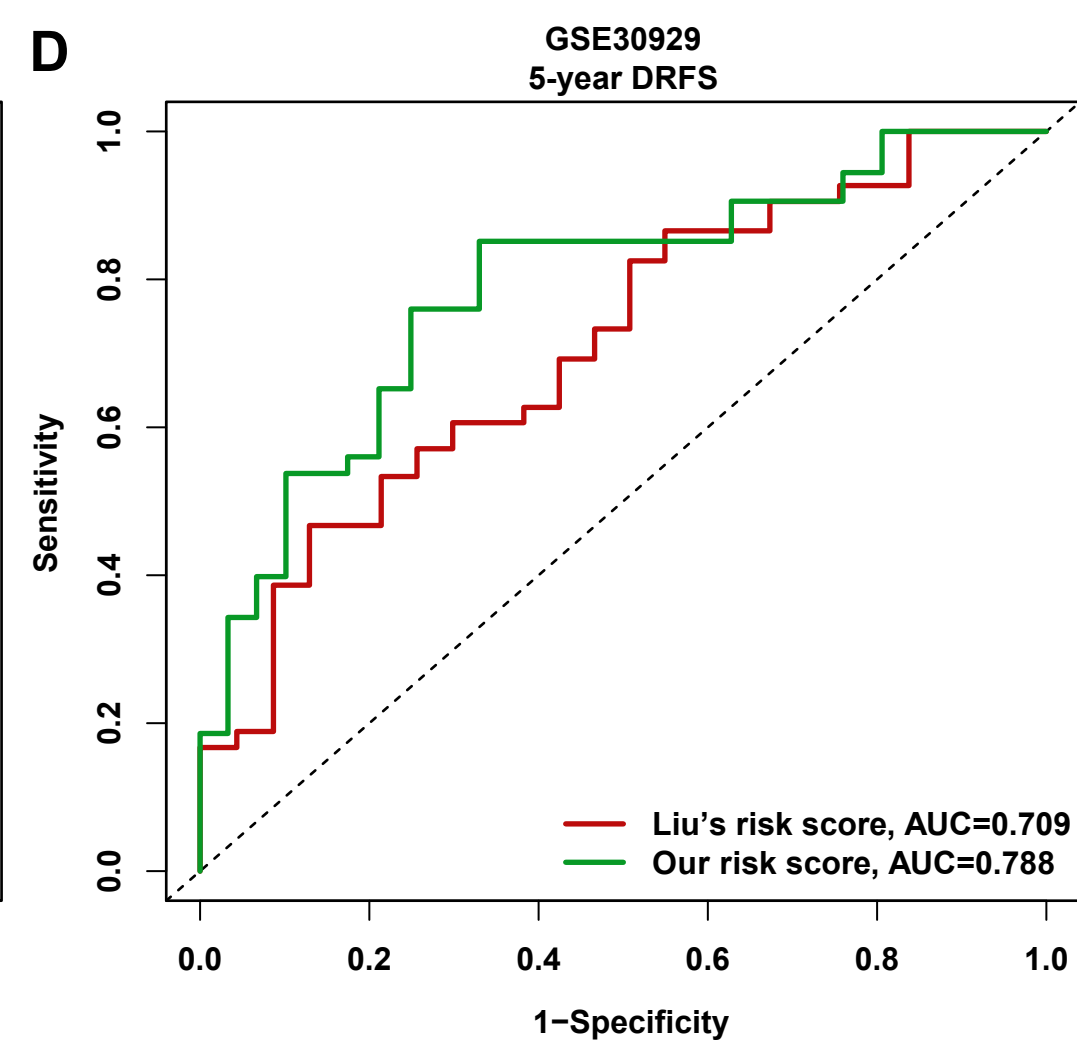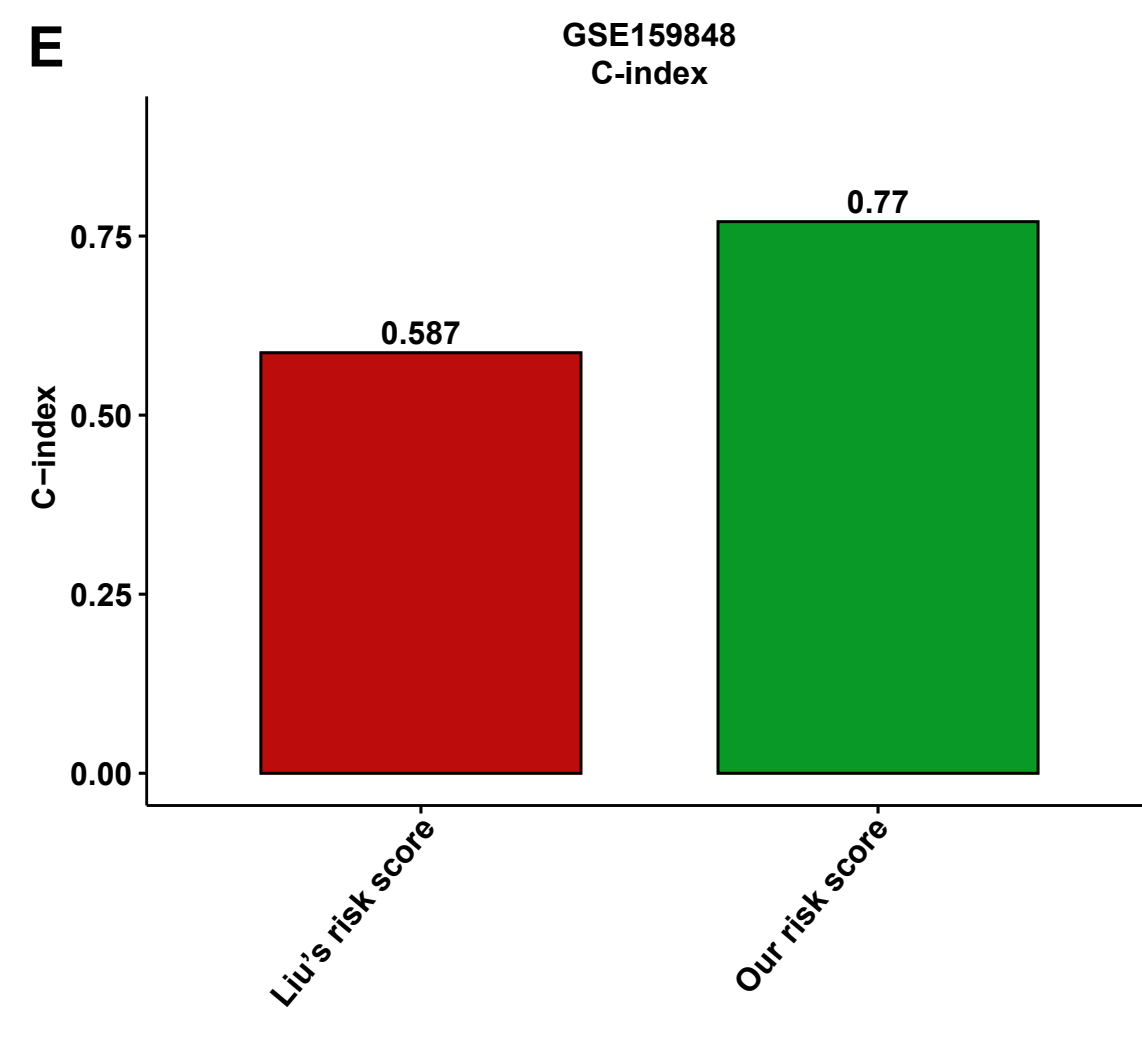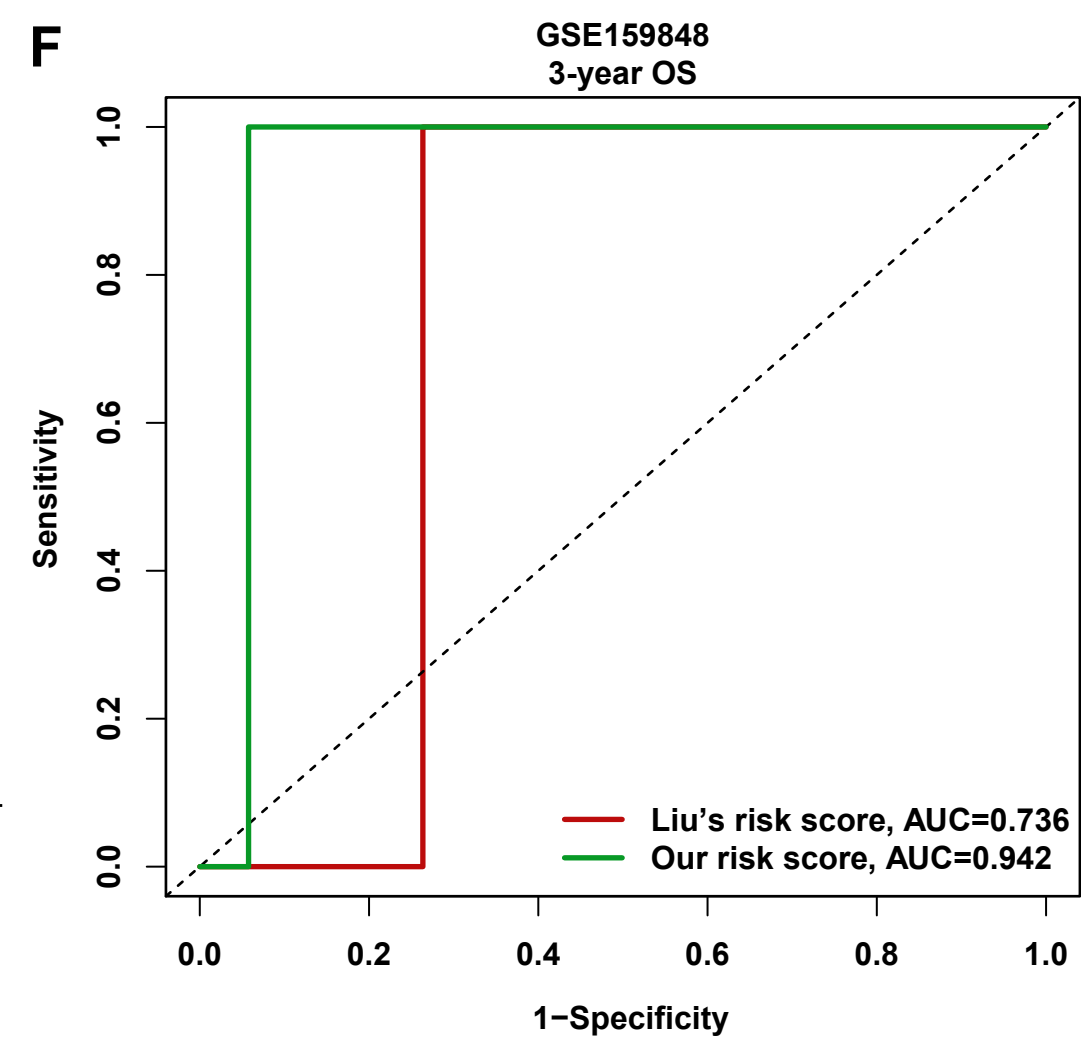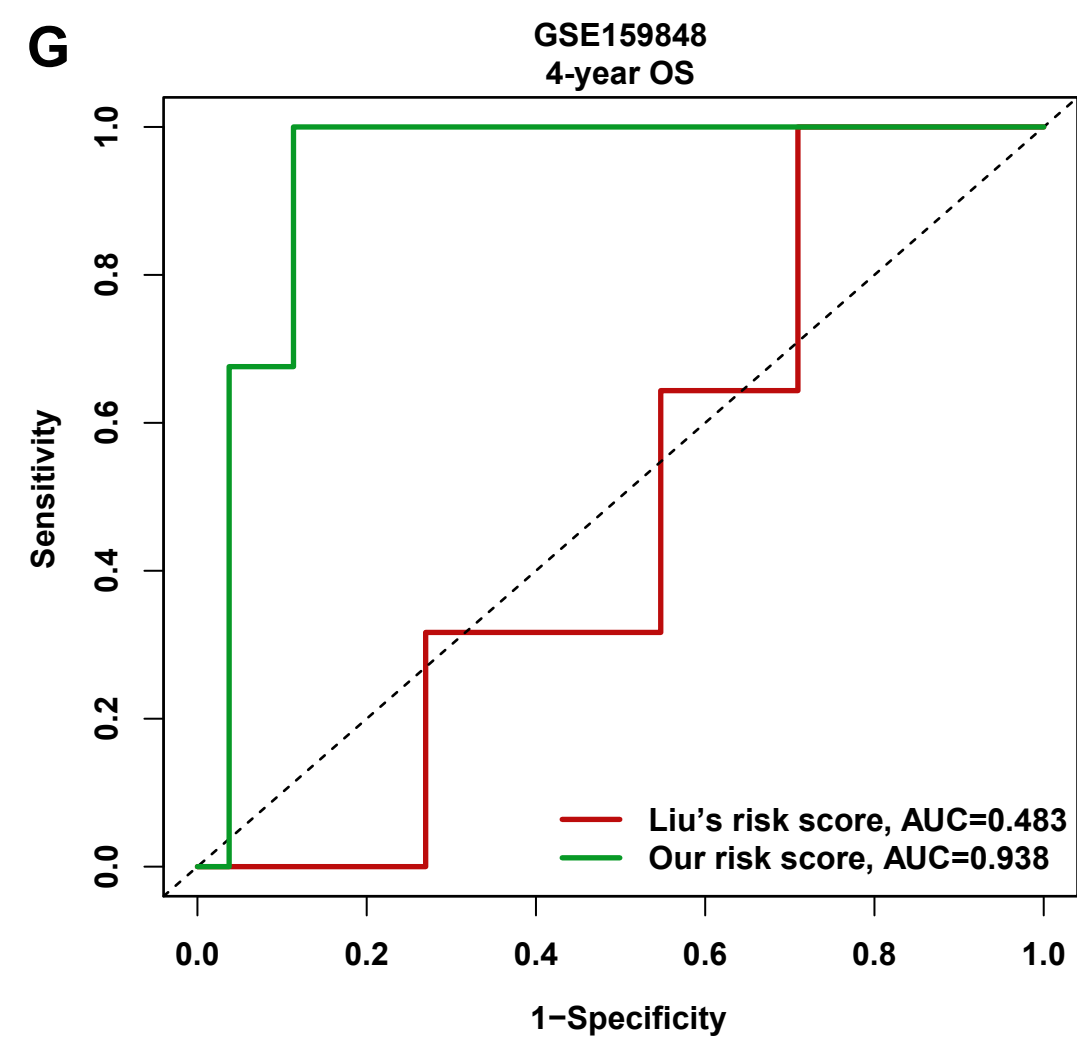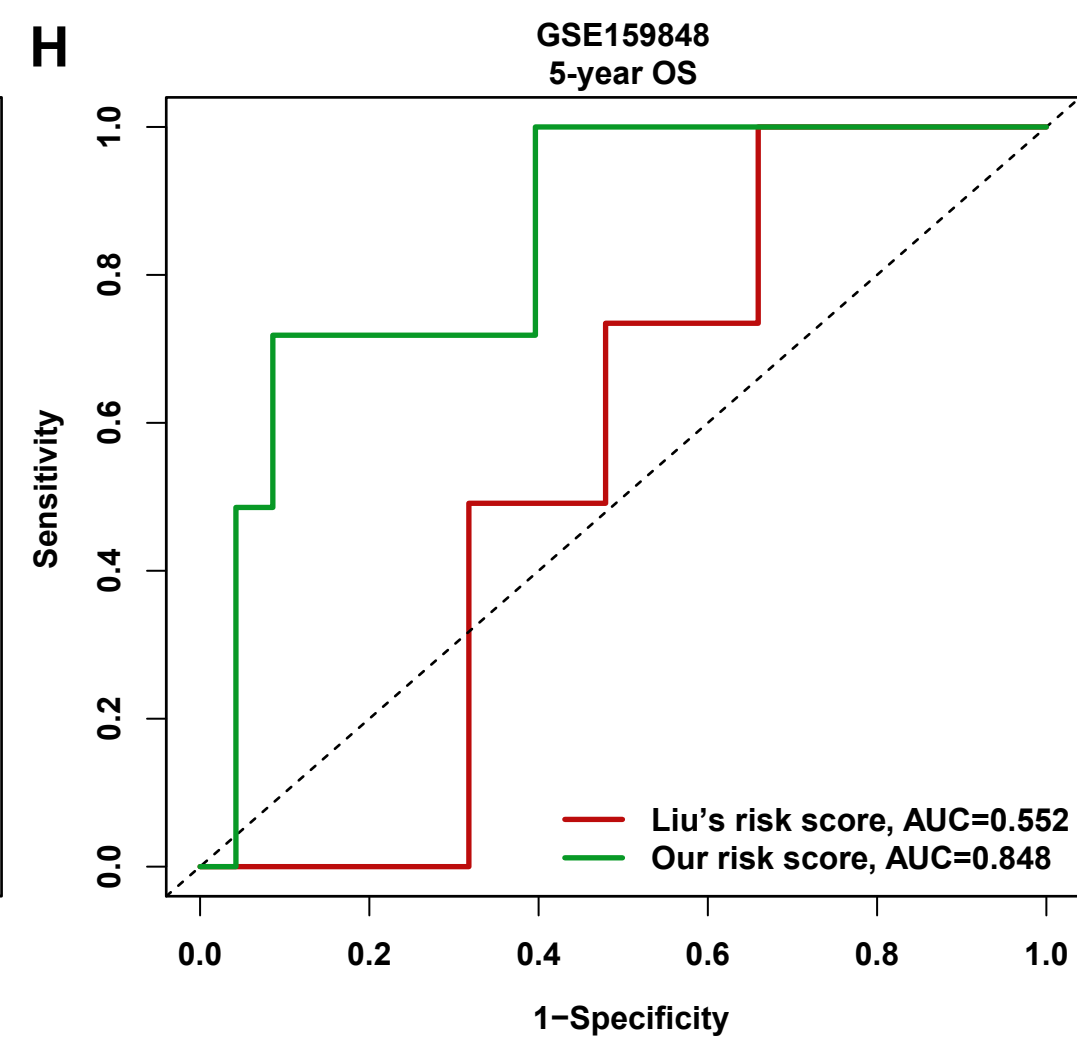

Supplement: Supplementary file 1 [file ijms-25-07792-s001.zip › Supplementary Figures/Figure S11.pdf]

A

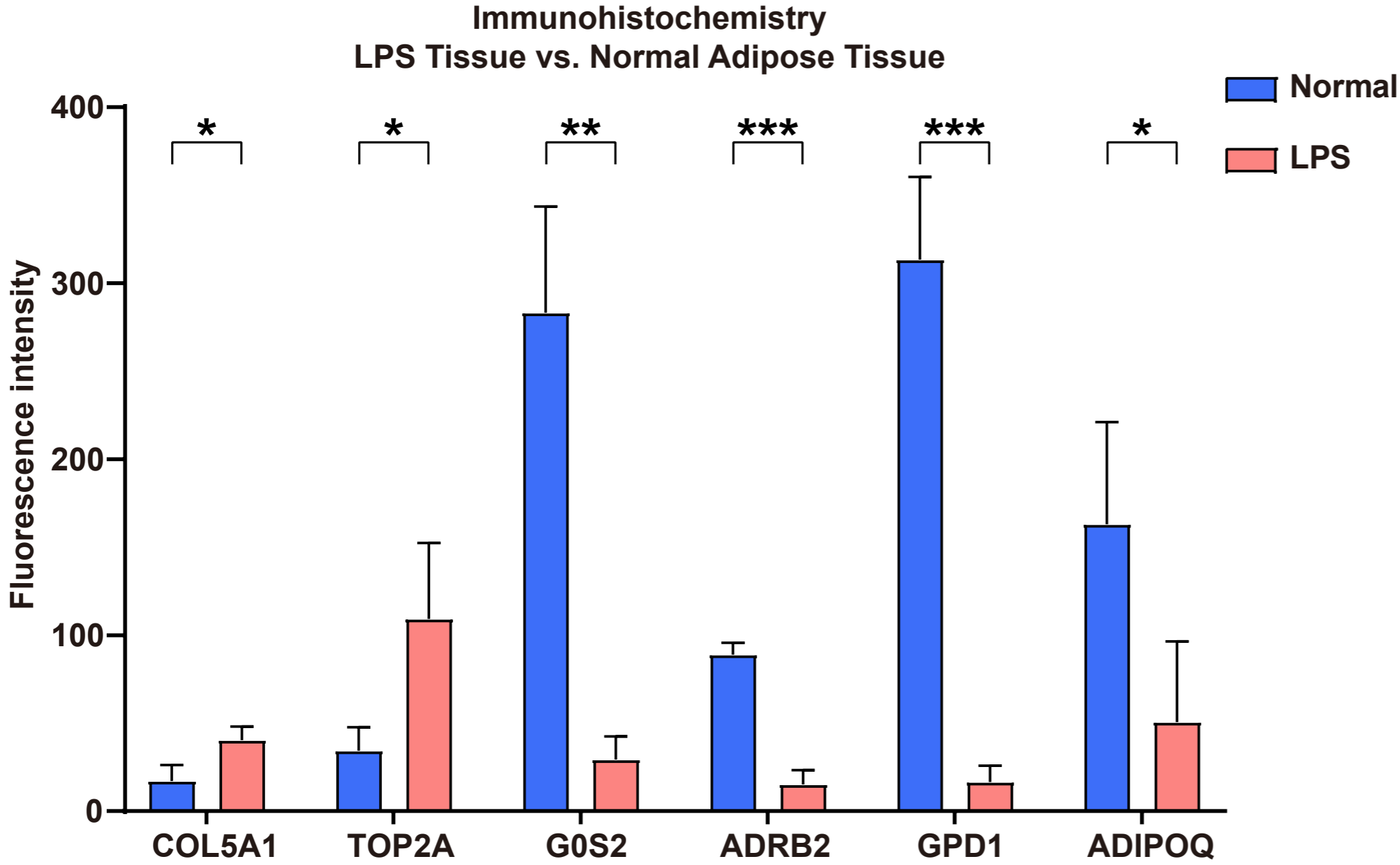

B

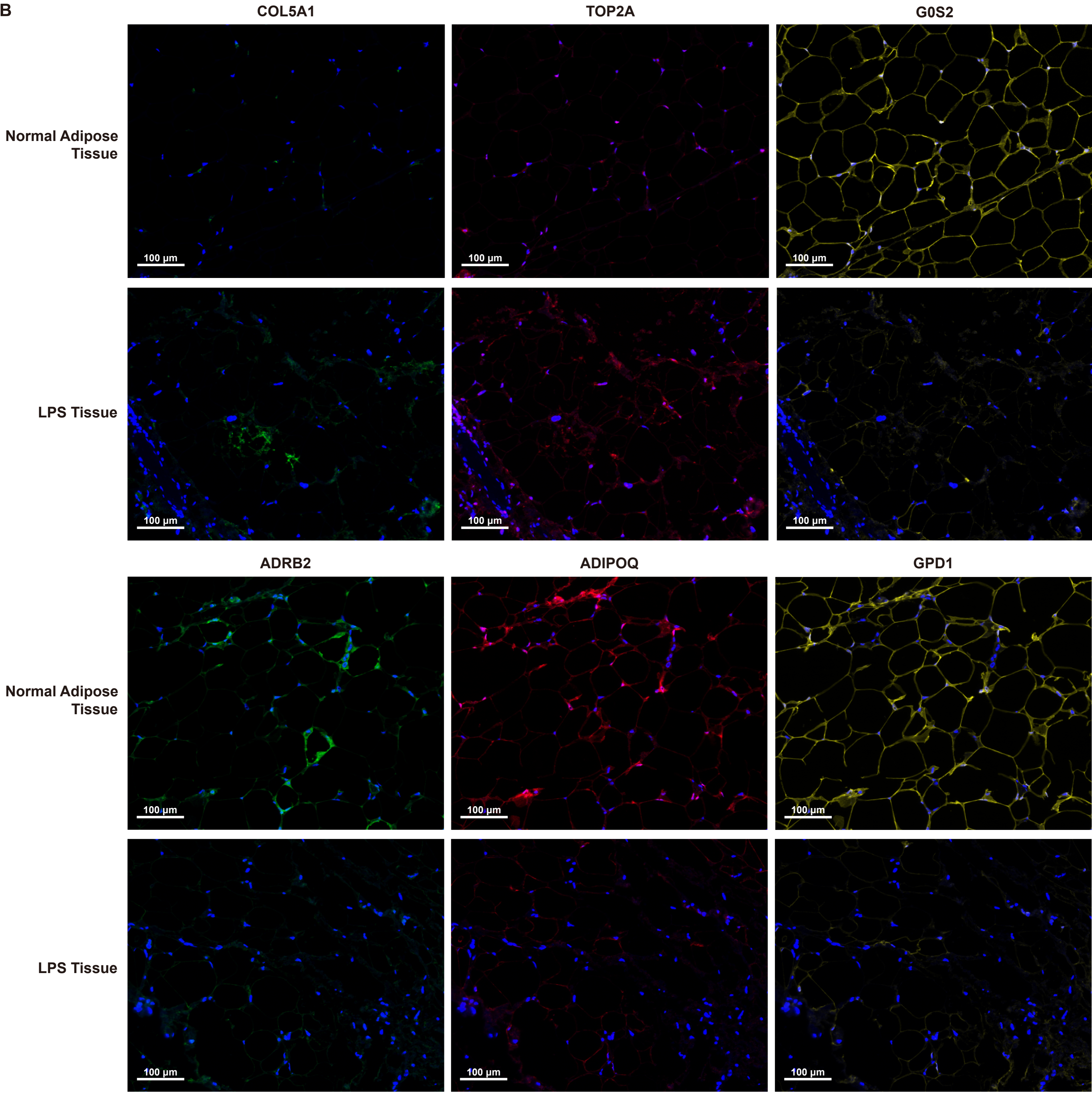

Supplement: Supplementary file 1 [file ijms-25-07792-s001.zip › Supplementary Figures/Figure S3_submit.pdf]

**A**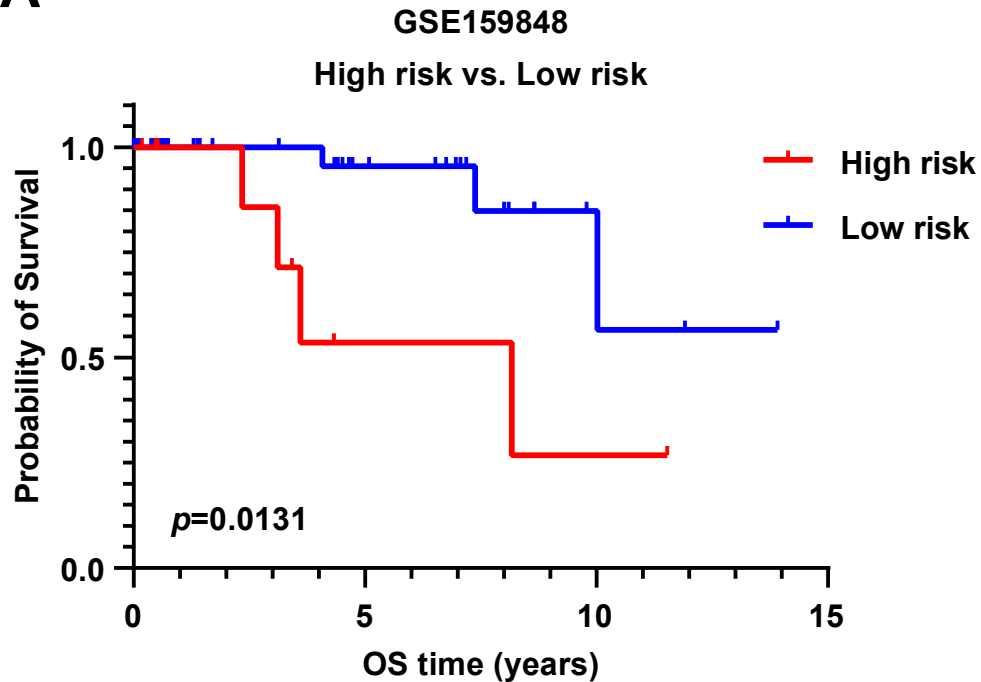**B**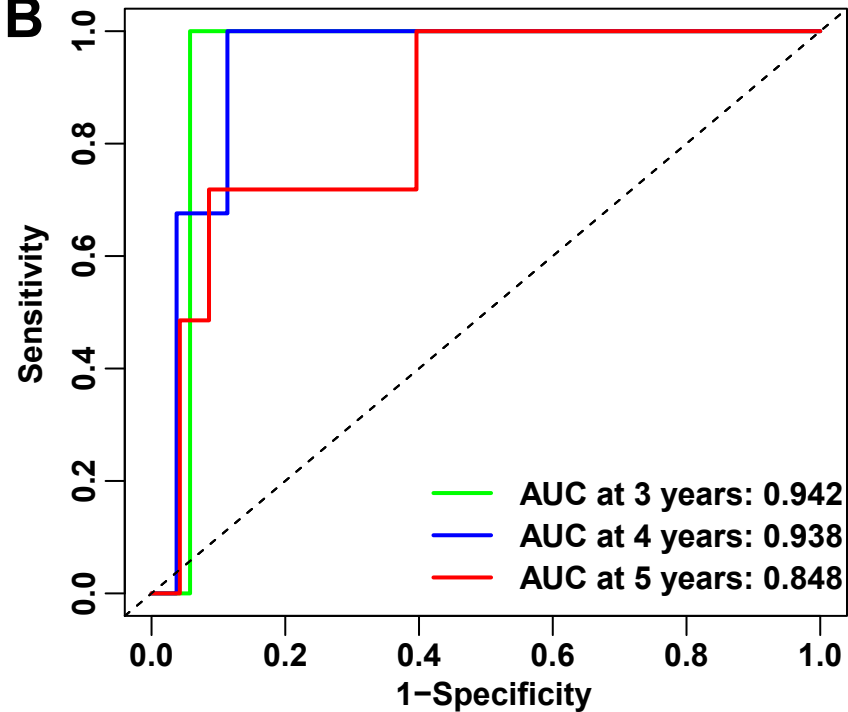

Supplement: Supplementary file 1 [file ijms-25-07792-s001.zip › Supplementary Figures/Figure S4.pdf]

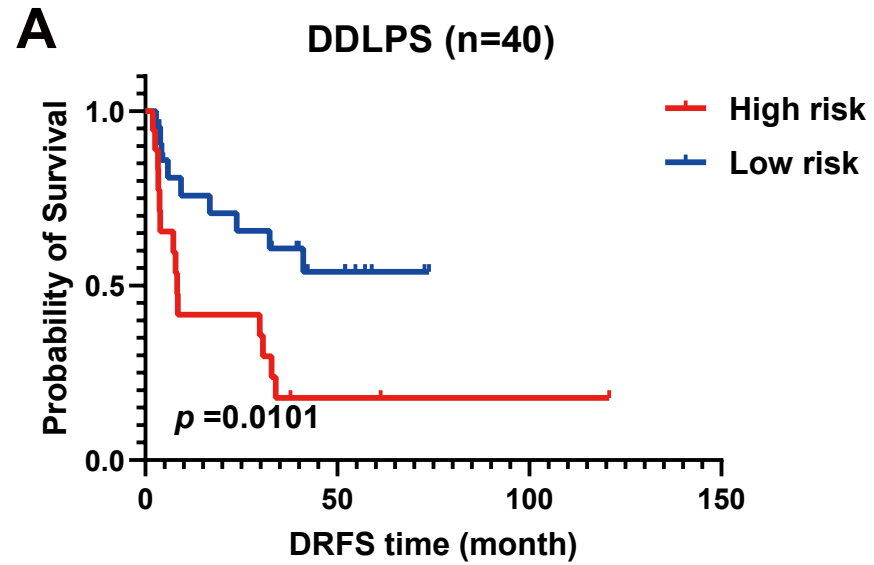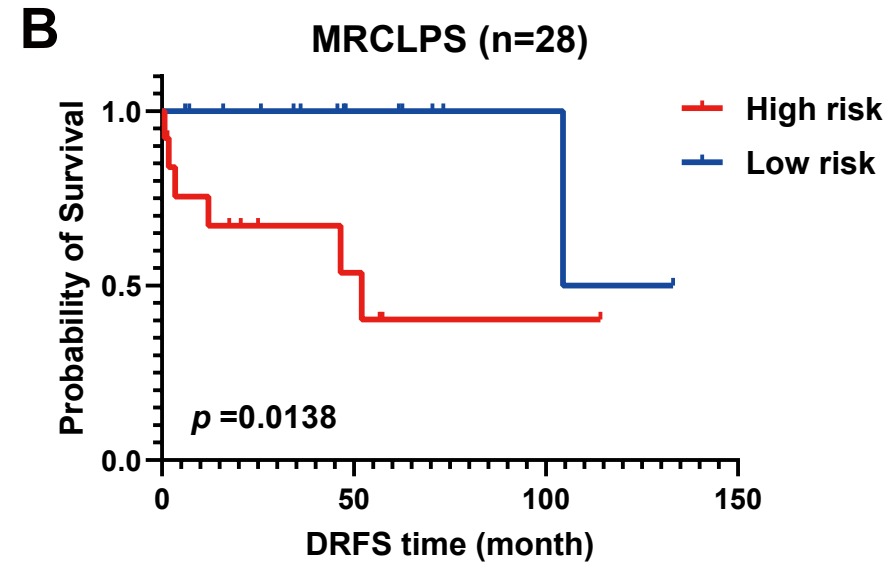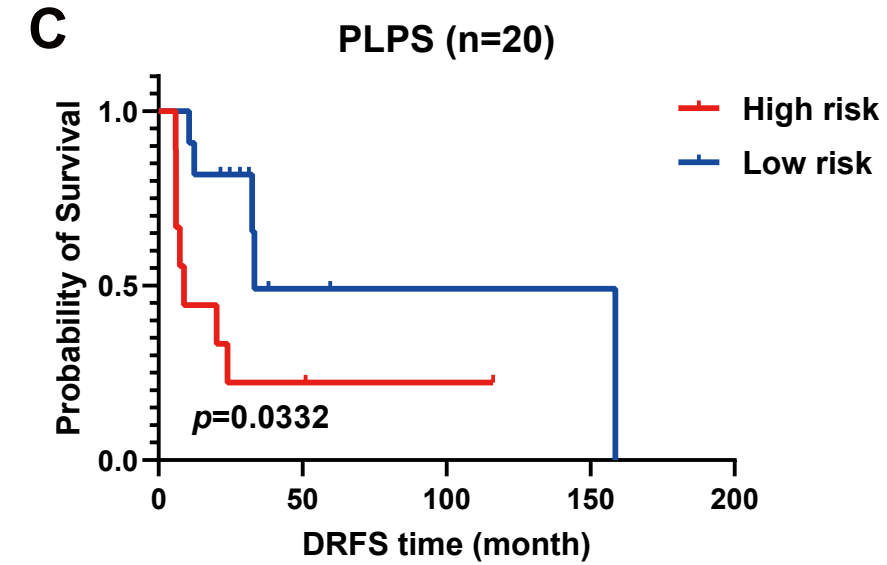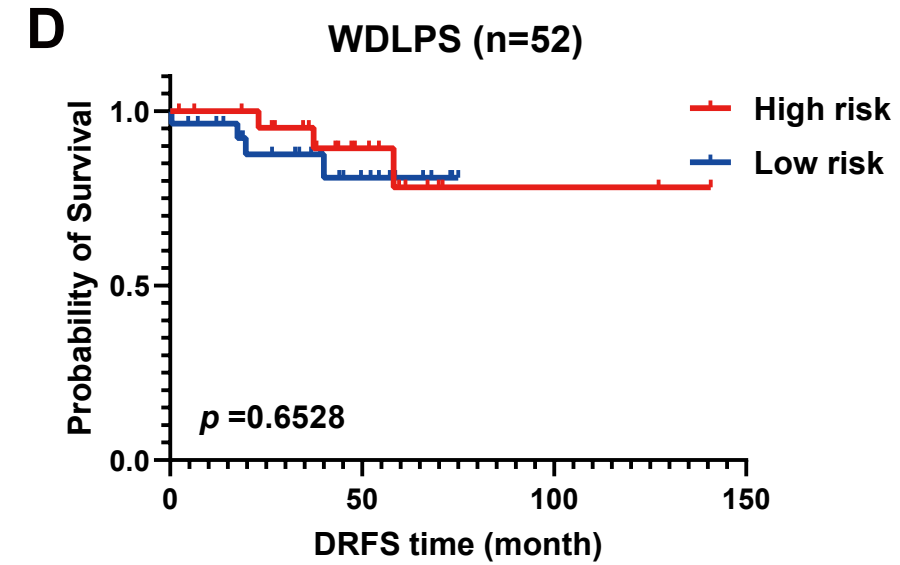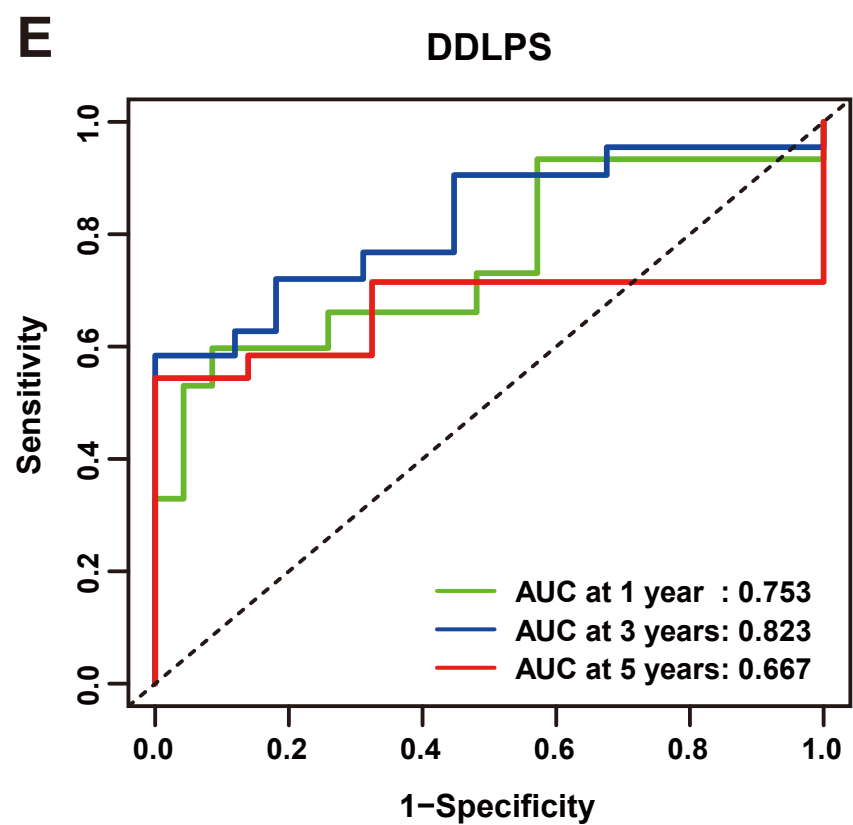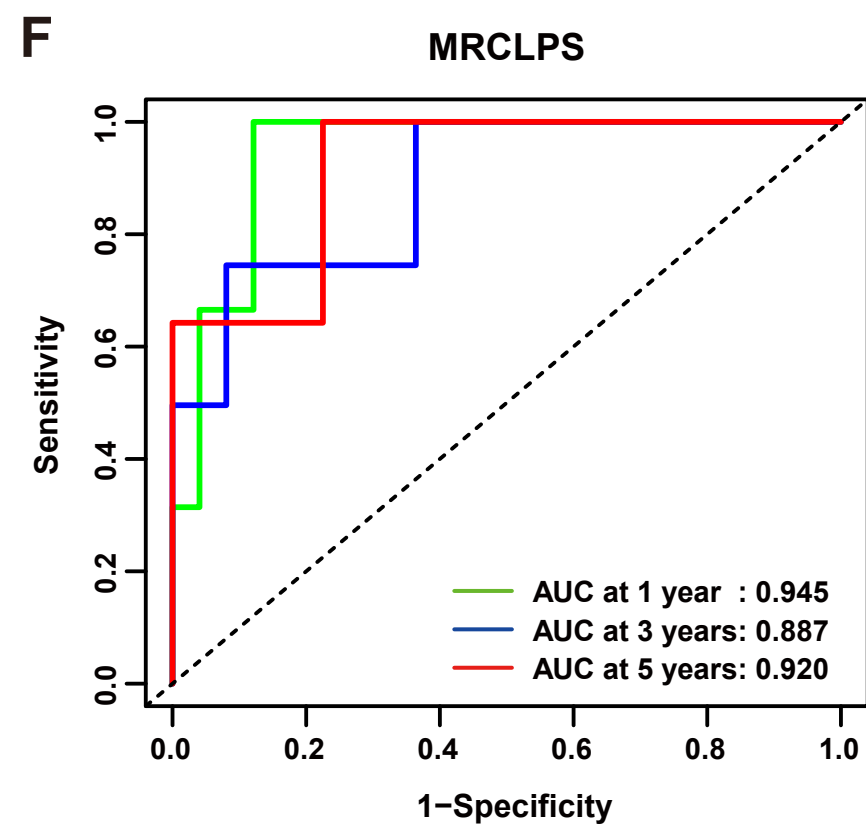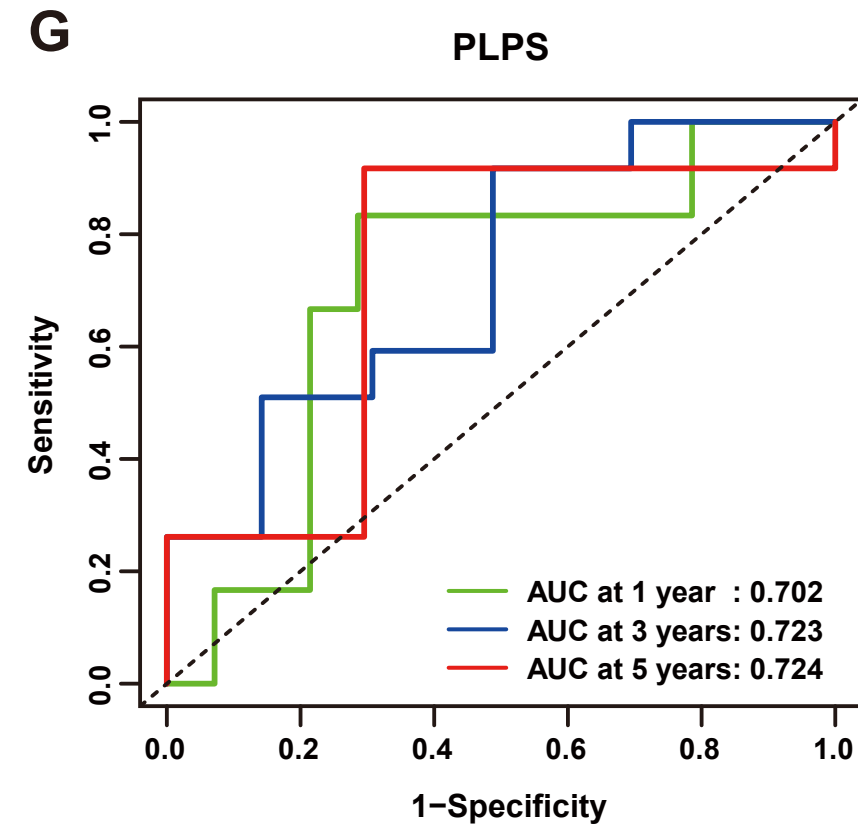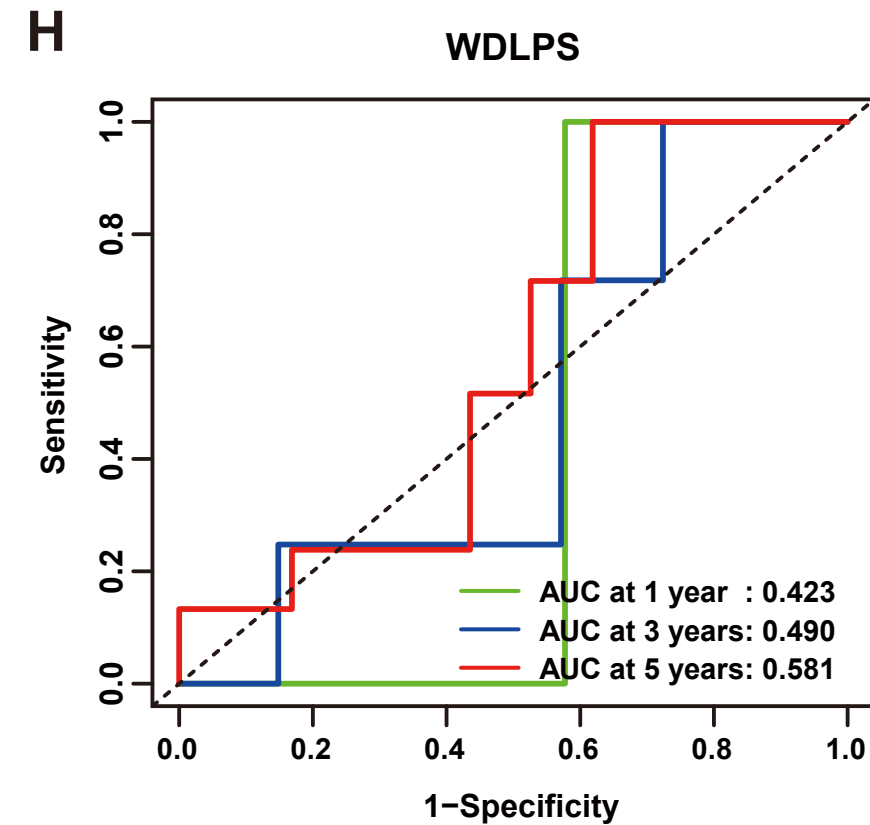

Supplement: Supplementary file 1 [file ijms-25-07792-s001.zip › Supplementary Figures/Figure S5.pdf]

**A**

Consensus matrix k=2

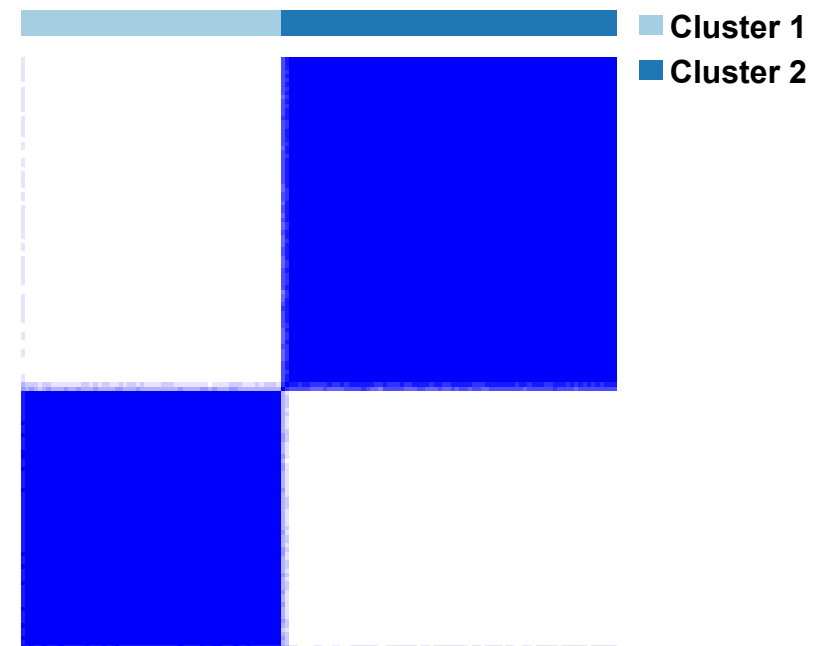**B**

Consensus CDF

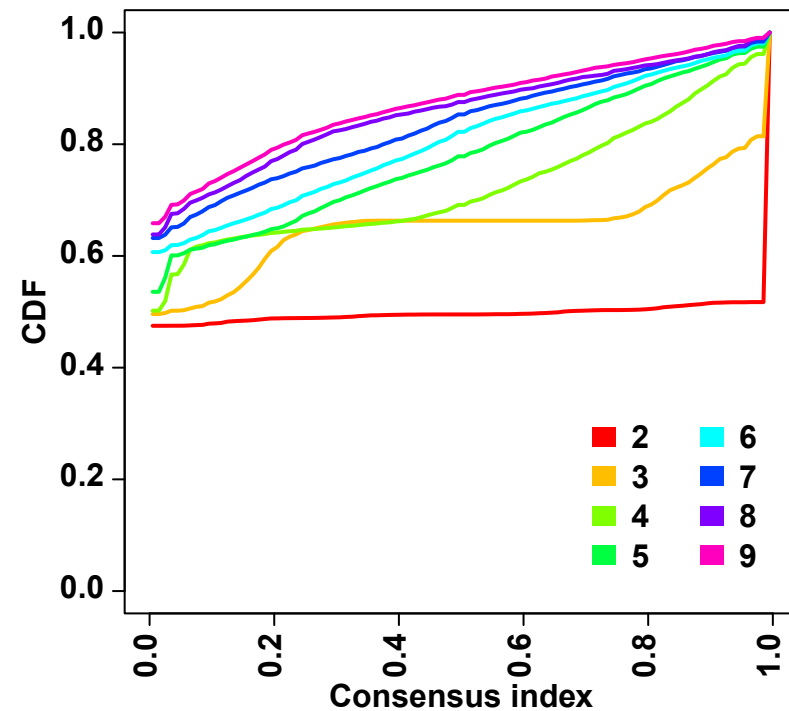**C**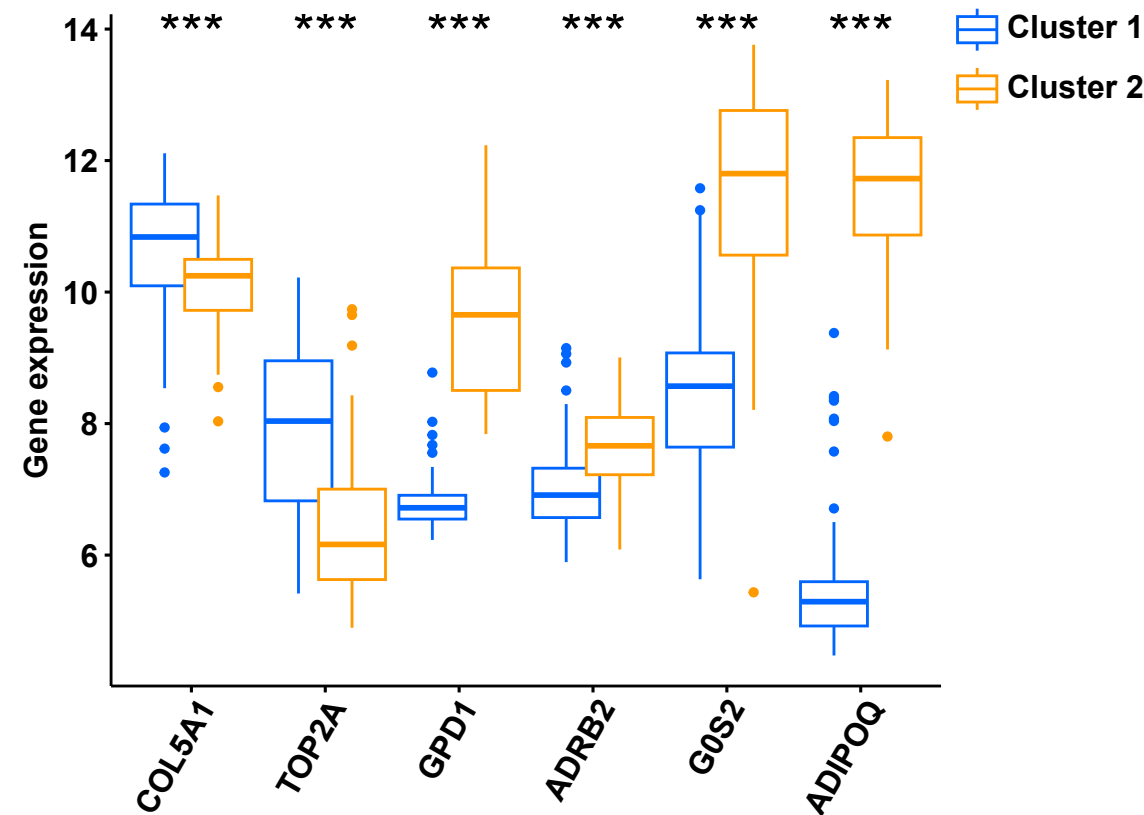**D**GSE30929  
Cluster 1 vs. Cluster 2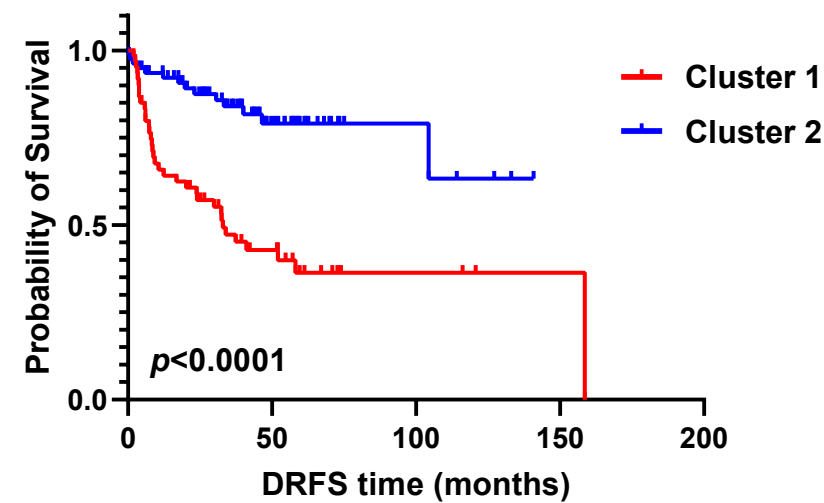**E**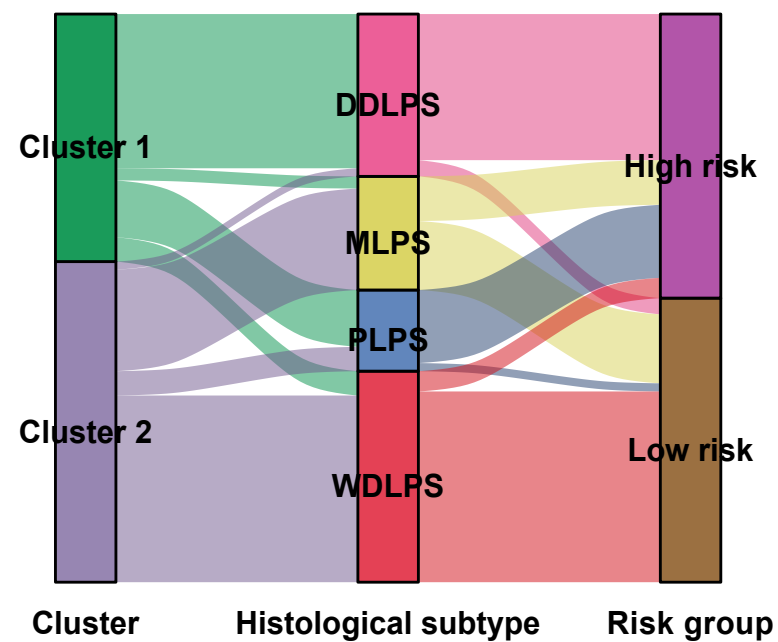

Supplement: Supplementary file 1 [file ijms-25-07792-s001.zip › Supplementary Figures/Figure S6.pdf]

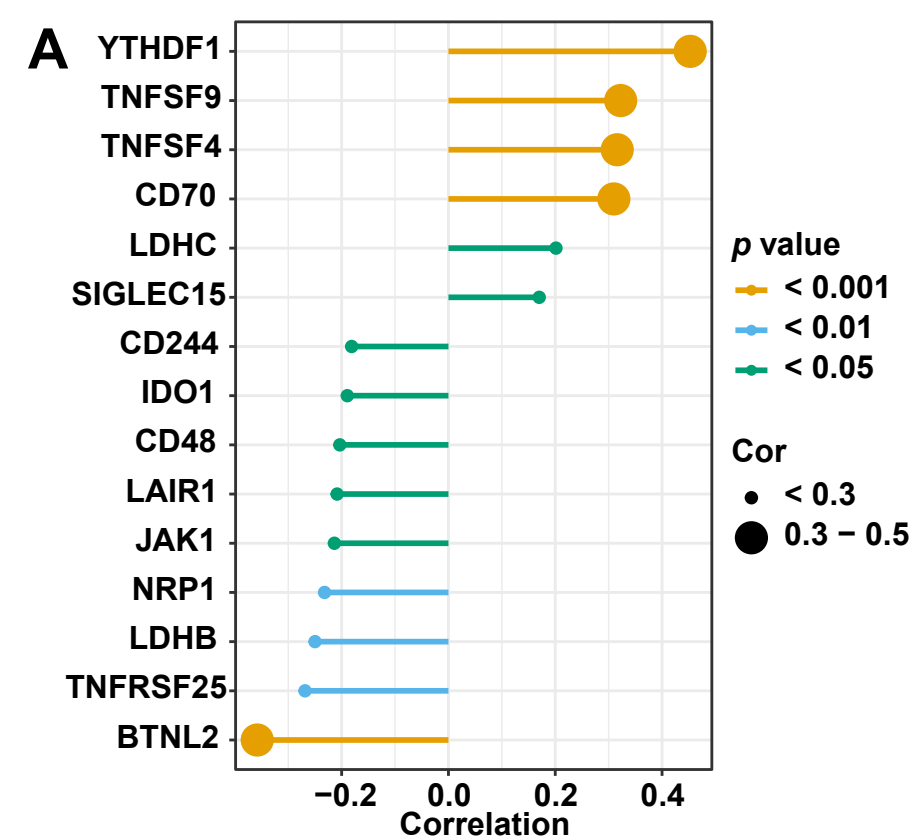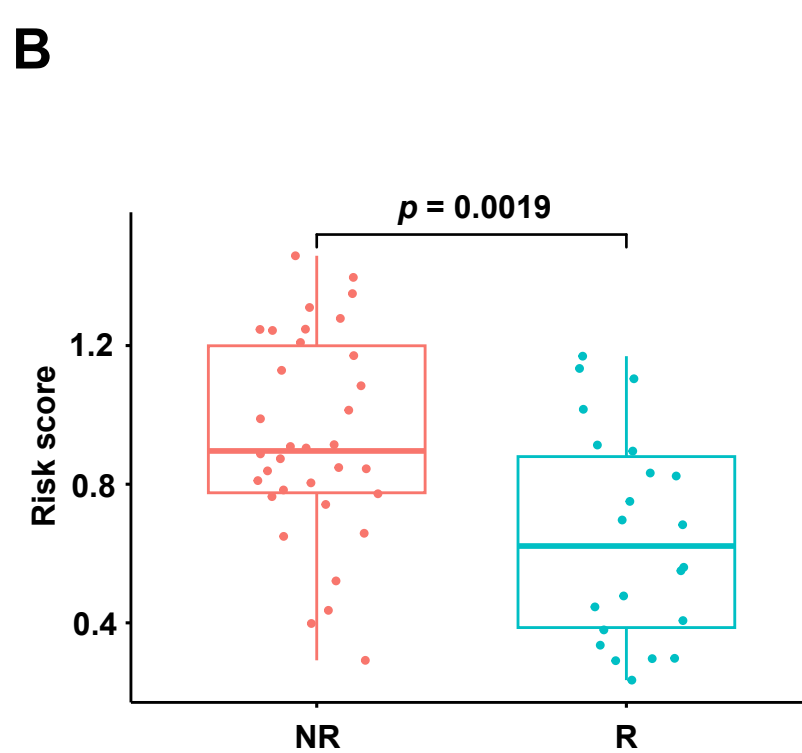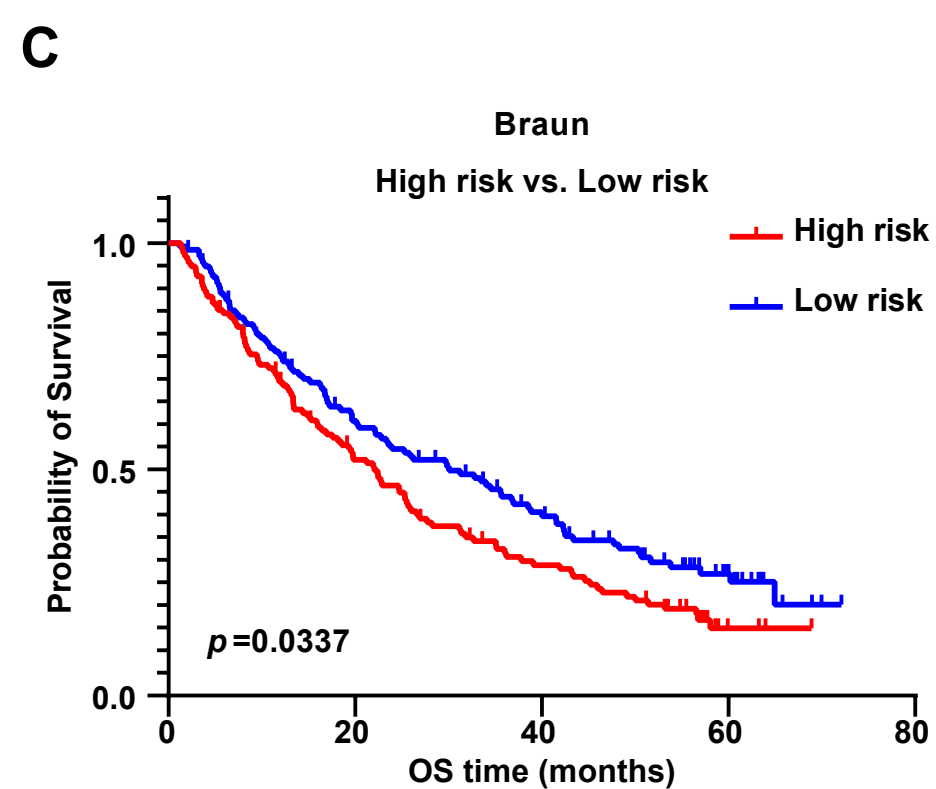

Supplement: Supplementary file 1 [file ijms-25-07792-s001.zip › Supplementary Figures/Figure S8.pdf]

**A**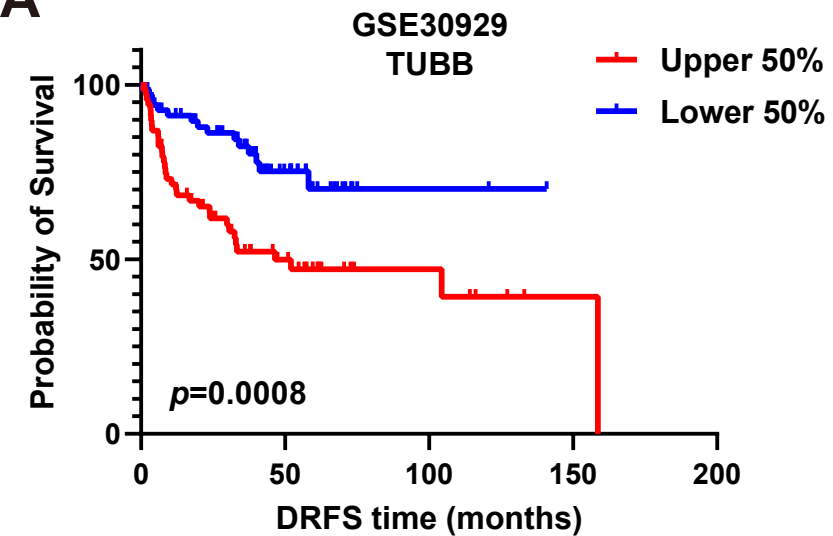**B**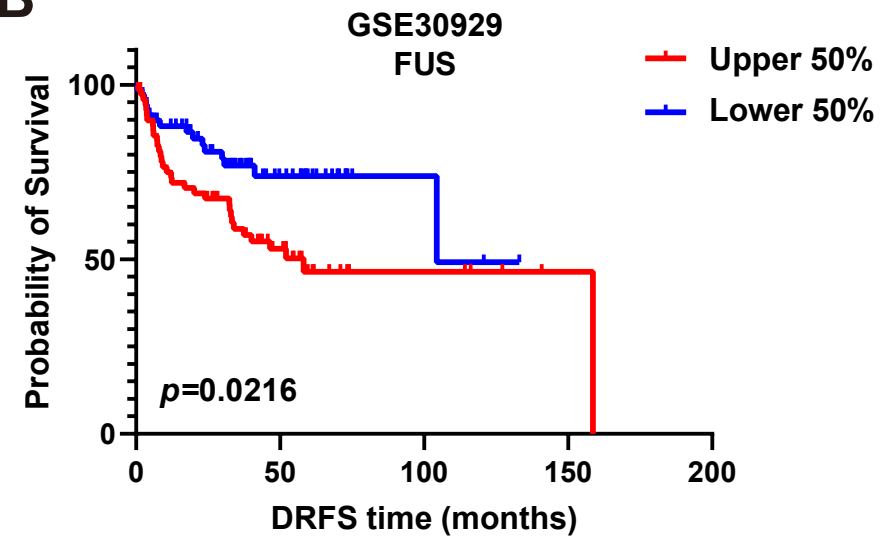**C**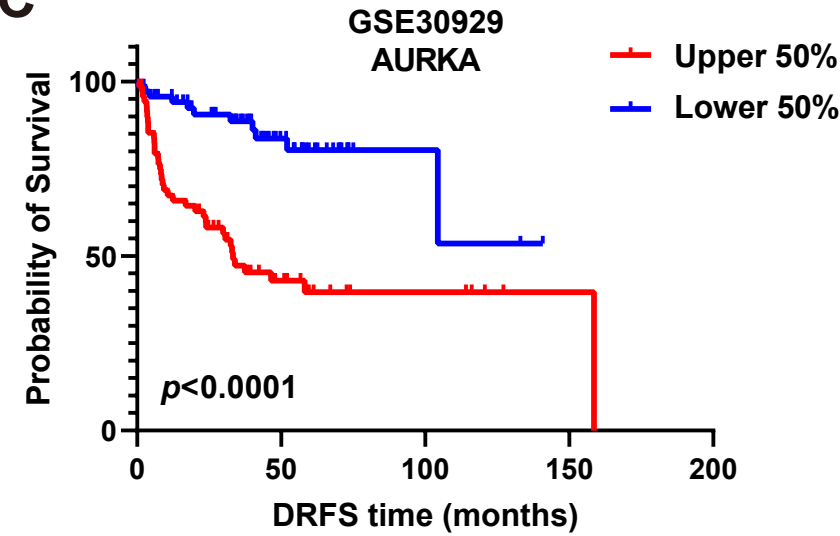**D**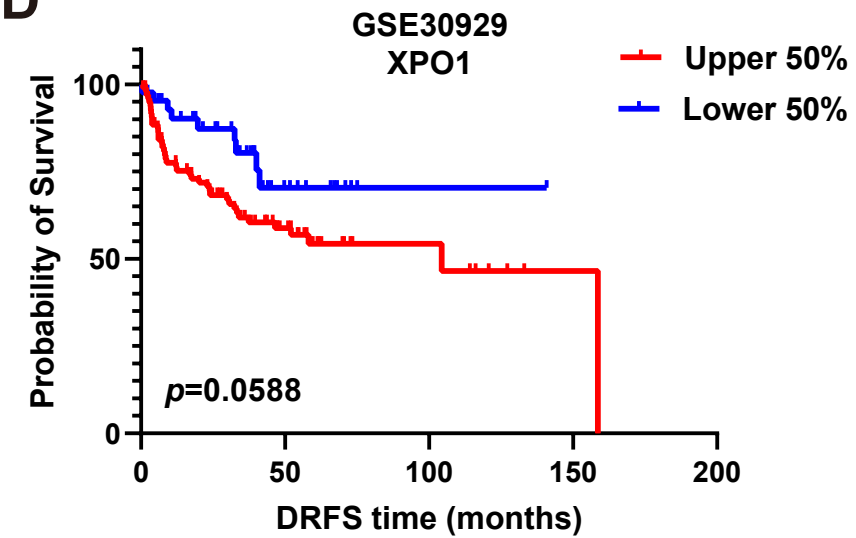**E**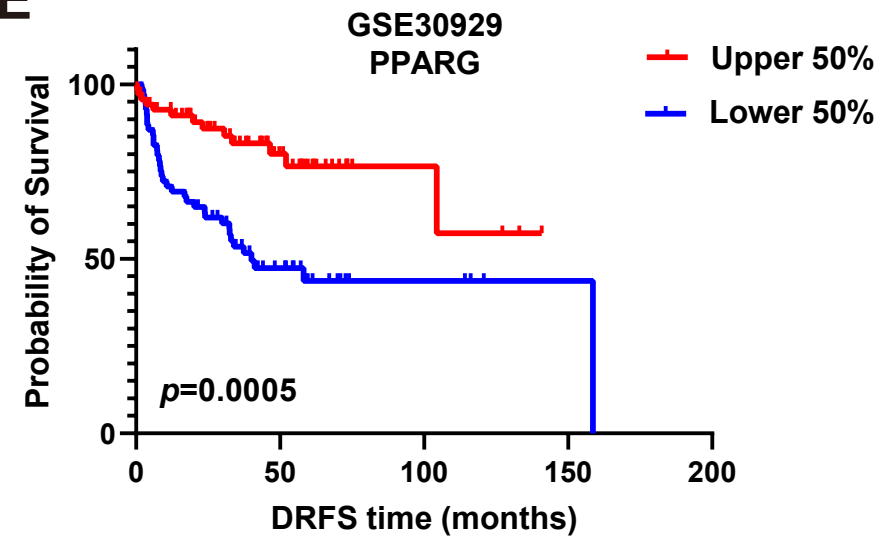

Supplement: Supplementary file 1 [file ijms-25-07792-s001.zip › Supplementary Figures/Figure S9.pdf]
