# Supplementary material for: Comprehensive Analysis of a Six-Gene Signature Predicting Survival and Immune Infiltration of Liposarcoma Patients and Deciphering Its Therapeutic Significance
Source: Int J Mol Sci. 2024 Jul 16;25(14):7792. doi: 10.3390/ijms25147792 (PMC11277418; doi:10.3390/ijms25147792)
Supplement: Supplementary file 1 [file Figure S2.pdf › Supplementary Figures/Figure S2.pdf]

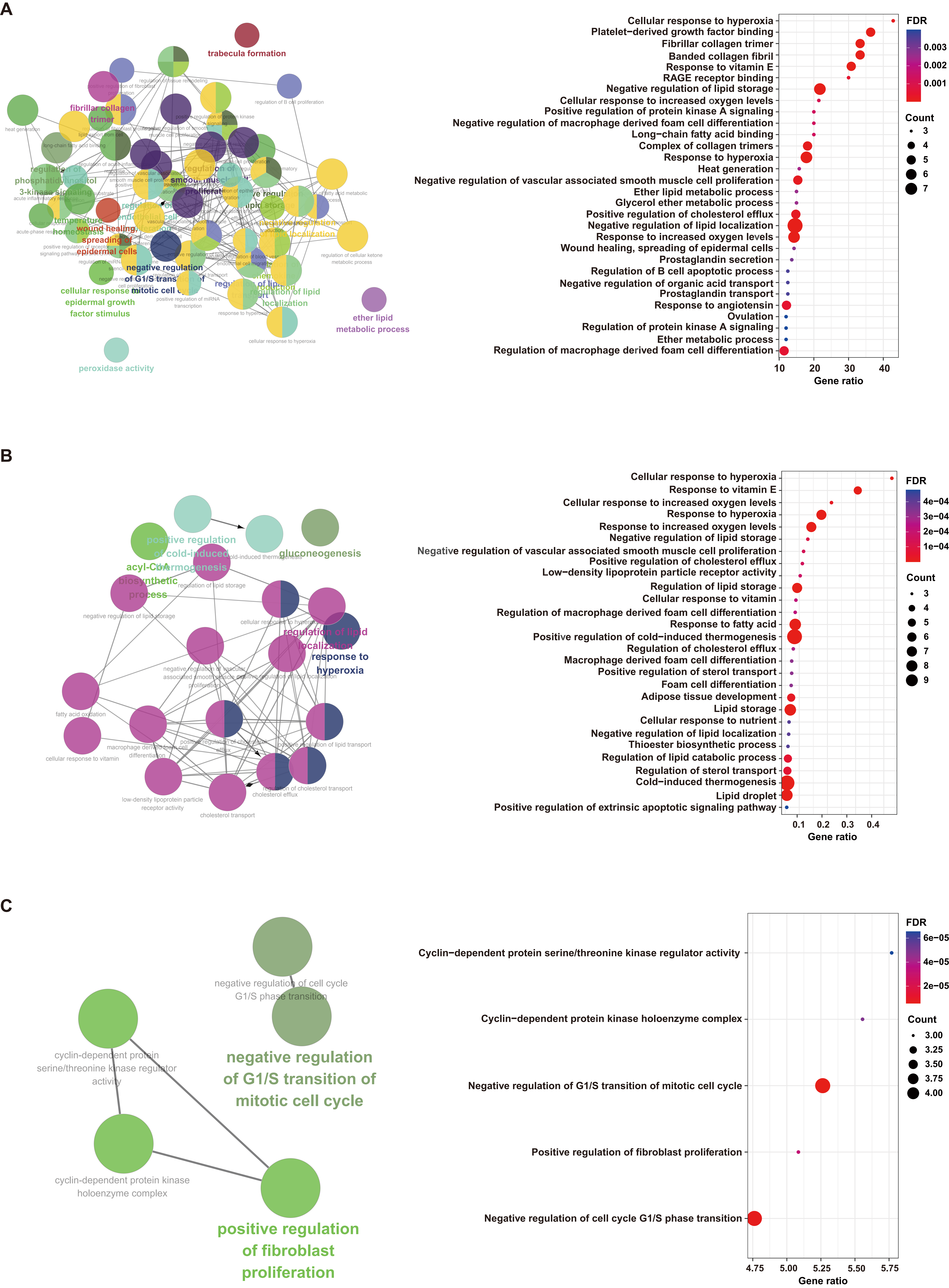
A
trabecula formation
fibrillar collagen trimer
ether lipid metabolic process
peroxidase activity
cellular response to epidermal growth factor stimulus
Cellular response to hyperoxia
Platelet−derived growth factor binding
Fibrillar collagen trimer
Banded collagen fibril
Response to vitamin E
RAGE receptor binding
Negative regulation of lipid storage
Cellular response to increased oxygen levels
Positive regulation of protein kinase A signaling
Negative regulation of macrophage derived foam cell differentiation
Long−chain fatty acid binding
Complex of collagen trimers
Response to hyperoxia
Heat generation
Negative regulation of vascular associated smooth muscle cell proliferation
Ether lipid metabolic process
Glycerol ether metabolic process
Positive regulation of cholesterol efflux
Negative regulation of lipid localization
Response to increased oxygen levels
Wound healing, spreading of epidermal cells
Prostaglandin secretion
Regulation of B cell apoptotic process
Negative regulation of organic acid transport
Prostaglandin transport
Response to angiotensin
Ovulation
Regulation of protein kinase A signaling
Ether metabolic process
Regulation of macrophage derived foam cell differentiation
10
20
30
40
Gene ratio
FDR
0.003
0.002
0.001
Count
3
4
5
6
7
B
gluconeogenesis
response to hyperoxia
regulation of lipid localization
negative regulation of lipid storage
regulation of lipid storage
fatty acid oxidation
cellular response to vitamin
low-density lipoprotein particle receptor activity
cholesterol transport
regulation of cholesterol transport
Cellular response to hyperoxia
Response to vitamin E
Cellular response to increased oxygen levels
Response to hyperoxia
Response to increased oxygen levels
Negative regulation of lipid storage
Negative regulation of vascular associated smooth muscle cell proliferation
Positive regulation of cholesterol efflux
Low−density lipoprotein particle receptor activity
Regulation of lipid storage
Cellular response to vitamin
Regulation of macrophage derived foam cell differentiation
Response to fatty acid
Positive regulation of cold−induced thermogenesis
Regulation of cholesterol efflux
Macrophage derived foam cell differentiation
Positive regulation of sterol transport
Foam cell differentiation
Adipose tissue development
Lipid storage
Cellular response to nutrient
Negative regulation of lipid localization
Thioester biosynthetic process
Regulation of lipid catabolic process
Regulation of sterol transport
Cold−induced thermogenesis
Lipid droplet
Positive regulation of extrinsic apoptotic signaling pathway
0.1
0.2
0.3
0.4
Gene ratio
FDR
4e−04
3e−04
2e−04
1e−04
Count
3
4
5
6
7
8
9
C
negative regulation of cell cycle G1/S phase transition
cyclin-dependent protein serine/threonine kinase regulator activity
negative regulation of G1/S transition of mitotic cell cycle
cyclin-dependent protein kinase holoenzyme complex
positive regulation of fibroblast proliferation
Cyclin−dependent protein serine/threonine kinase regulator activity
Cyclin−dependent protein kinase holoenzyme complex
Negative regulation of G1/S transition of mitotic cell cycle
Positive regulation of fibroblast proliferation
Negative regulation of cell cycle G1/S phase transition
4.75
5.00
5.25
5.50
5.75
Gene ratio
FDR
6e−05
4e−05
2e−05
Count
3.00
3.25
3.50
3.75
4.00
