# Supplementary material for: Comprehensive Analysis of a Six-Gene Signature Predicting Survival and Immune Infiltration of Liposarcoma Patients and Deciphering Its Therapeutic Significance
Source: Int J Mol Sci. 2024 Jul 16;25(14):7792. doi: 10.3390/ijms25147792 (PMC11277418; doi:10.3390/ijms25147792)
Supplement: Supplementary file 1 [file Figure S7.pdf › Supplementary Figures/Figure S7.pdf]

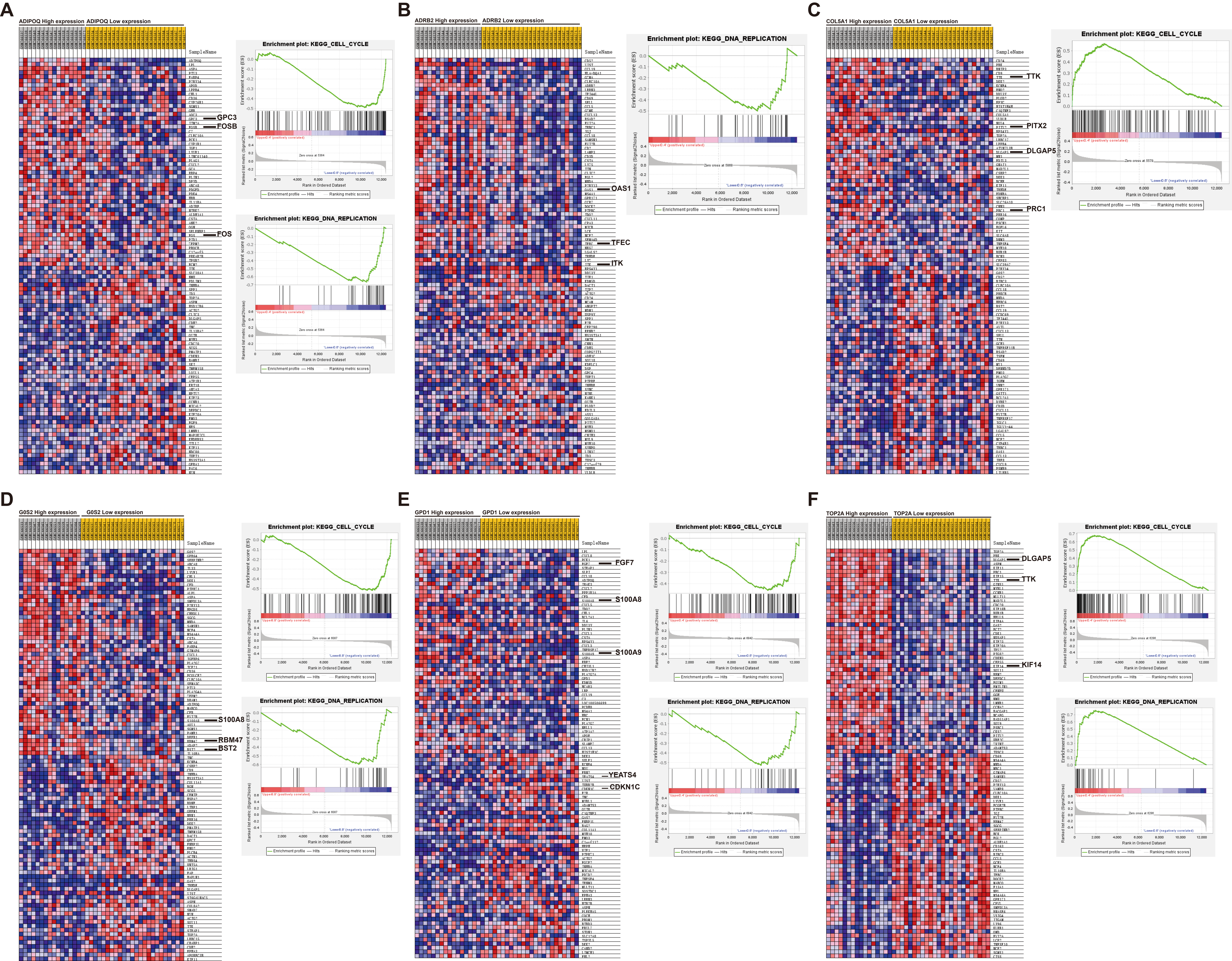
A
ADIPOQ High expression
ADIPOQ Low expression
GPC3
FOSB
FOS
Enrichment plot: KEGG_CELL_CYCLE
Enrichment plot: KEGG_DNA_REPLICATION
B
ADRB2 High expression
ADRB2 Low expression
OAS1
TFEC
ITK
Enrichment plot: KEGG_DNA_REPLICATION
C
COL5A1 High expression
COL5A1 Low expression
TTK
PITX2
DLGAP5
PRC1
Enrichment plot: KEGG_CELL_CYCLE
D
G0S2 High expression
G0S2 Low expression
S100A8
RBM47
BST2
Enrichment plot: KEGG_CELL_CYCLE
Enrichment plot: KEGG_DNA_REPLICATION
E
GPD1 High expression
GPD1 Low expression
FGF7
S100A8
S100A9
YEATS4
CDKN1C
Enrichment plot: KEGG_CELL_CYCLE
Enrichment plot: KEGG_DNA_REPLICATION
F
TOP2A High expression
TOP2A Low expression
DLGAP5
TTK
KIF14
Enrichment plot: KEGG_CELL_CYCLE
Enrichment plot: KEGG_DNA_REPLICATION
